# UBE2V1 Promotes Hepatocellular Carcinoma Progression by Forming a Positive Feedback Loop with HIF-1α

**DOI:** 10.34133/research.1041

**Published:** 2025-12-23

**Authors:** Zibo Yuan, Sipin Hu, Qingwei Zhu, Yuliang Fang, Xin Liu, Shuangshuang Li, Xiaoge Hu, Kangsheng Tu, Qiuran Xu, Dongsheng Huang, Di Cui

**Affiliations:** ^1^ General Surgery, Cancer Center, Department of Gastrointestinal and Pancreatic Surgery, Zhejiang Provincial People’s Hospital (Affiliated People’s Hospital), Hangzhou Medical College, Hangzhou, Zhejiang, China.; ^2^ The Qingdao Medical College of Qingdao University, Qingdao, Shandong, China.; ^3^ Zhejiang Key Laboratory of Tumor Molecular Diagnosis and Individualized Medicine, Zhejiang Provincial People’s Hospital (Affiliated People’s Hospital), Hangzhou Medical College, Hangzhou, Zhejiang, China.; ^4^ The Second Clinical Medical College of Zhejiang Chinese Medical University, Hangzhou, Zhejiang, China.; ^5^ Department of Burn and Plastic Surgery, Shandong Provincial Hospital Affiliated to Shandong First Medical University, Jinan, China.; ^6^ School of Clinical Medicine, Hangzhou Medical College, Hangzhou, Zhejiang, China.; ^7^ Department of Hepatobiliary Surgery, The First Affiliated Hospital of Xi’an Jiaotong University, Xi’an, Shaanxi, China.

## Abstract

Hepatocellular carcinoma (HCC) is characterized by a profoundly hypoxic microenvironment, which drives tumor aggressiveness and poor clinical outcomes. However, the precise regulatory mechanisms through which hypoxia promotes HCC progression remain incompletely understood. Here, we identified ubiquitin conjugating enzyme E2 variant 1 (*UBE2V1*) as a novel hypoxia-responsive gene that is transcriptionally activated by hypoxia-inducible factor-1α (HIF-1α) through direct binding to a hypoxia-response element located between −208 and −201 bp in the *UBE2V1* promoter. Overexpression of UBE2V1 was frequently detected in HCC tissues and correlated strongly with advanced tumor stage and unfavorable patient prognosis. Moreover, UBE2V1 facilitated the proliferation and migration of HCC cells. Further investigation revealed that up-regulated UBE2V1 competes with HIF-1α for binding to the β-domain of von Hippel–Lindau (VHL) protein and, in complex with UBE2S, catalyzes K11/K48-linked ubiquitination at VHL K196, leading to its proteasomal degradation. This disruption of VHL function attenuates HIF-1α ubiquitination and degradation, resulting in sustained HIF-1α stabilization, increased nuclear accumulation, and enhanced transcriptional activity. Consistent with these findings, genetic knockdown of *UBE2V1* or pharmacological inhibition of HIF-1α markedly suppresses HCC tumorigenesis and metastasis in vivo. Altogether, our study unveils a previously unrecognized HIF-1α–UBE2V1 positive feedback loop that is self-reinforcing and critically sustains the hypoxic microenvironment to drive HCC progression, highlighting UBE2V1 as both a promising prognostic biomarker and a compelling therapeutic target for HCC.

## Introduction

Hepatocellular carcinoma (HCC) is the sixth most commonly diagnosed cancer worldwide and accounts for the third highest number of cancer-related deaths [1]. Although substantial progress has been made in treatment strategies including surgical resection, liver transplantation, and targeted therapies, the 5-year overall survival (OS) rate for patients with HCC is still less than 20% [1,2]. This poor prognosis is primarily due to the silent progression of HCC during its early stages. In the absence of specific and sensitive early diagnostic biomarkers, only approximately 30% of patients are diagnosed at a stage amenable to curative treatment, while the remaining 70% rely on systemic therapies, which often provide suboptimal survival benefits due to multiple clinical and biological constraints [3]. Therefore, elucidating the key molecular mechanisms driving HCC progression and identifying molecular biomarkers for early diagnosis and prognosis assessment are imperative for the development of more effective therapeutic strategies.

Hypoxia is a hallmark of the HCC tumor microenvironment, strongly associated with cancer progression, metastasis, angiogenesis, therapy resistance, and poor clinical outcomes [4–7]. Hypoxia-inducible factor-1α (HIF-1α), a key transcription factor involved in the cellular response to hypoxia, is precisely controlled mainly via the ubiquitin–proteasome system (UPS). Under normoxic conditions, HIF-α is hydroxylated on prolyl residues by PHD enzymes and then marked for K48-linked ubiquitination by the von Hippel–Lindau (VHL)-associated E3 ubiquitin ligase complex, leading to its degradation via the proteasome [8]. During hypoxia, HIF-1α escapes degradation to form transcriptional complexes and activates gene expression by binding to hypoxia-response elements (HREs, 5′-RCGTG-3′) in the promoter of target genes [9]. Aberrant stabilization and activation of HIF-1α, frequently observed in HCC patients with poor prognosis, drives tumor progression by transcriptionally activating downstream targets involved in angiogenesis (e.g., vascular endothelial growth factor [VEGF]), metabolic reprogramming (e.g., glucose transporter 1 and 3 [GLUT1 and GLUT3]), and metastasis (e.g., twist family BHLH transcription factor [TWIST]) [10–13]. Notably, HIF-1α accumulation is not solely oxygen-dependent but is also closely associated with dysregulation of the UPS [14–16]. However, the complex regulatory network governing HIF-1α ubiquitination has yet to be fully elucidated.

Ubiquitin conjugating enzyme E2 variant 1 (UBE2V1), a core component of the ubiquitin-conjugating enzyme family, plays a critical role in the UPS and has been implicated in autophagy, protein homeostasis, and the activation of signaling pathways that drive tumor progression and reproductive aging. Since the UBE2V1 protein lacks intrinsic ubiquitin ligase activity, it primarily performs its biological functions by forming a complex with UBC13. UBE2V1 promotes UBC13-mediated ubiquitination and subsequent degradation of Sirt1, thereby suppressing histone H4 lysine 16 acetylation and epigenetically down-regulating autophagy-related gene expression in renal cancer [17]. The UBE2V1–UBC13 heterodimer mediates K63-linked ubiquitination of TRAF6, leading to IKK complex activation and subsequent NF-κB nuclear translocation [18]. Moreover, UBE2V1 contributes to the formation of ubiquitinated protein aggregates, which can impair mitochondrial function and lead to developmental defects [19]. However, its biological function in HCC, especially within the hypoxic tumor microenvironment, is still not well characterized.

In this study, we demonstrated that the hypoxic tumor microenvironment induces the up-regulation of UBE2V1 in HCC, mediated through direct transcriptional activation by HIF-1α binding to an HRE in the *UBE2V1* promoter region. UBE2V1 was frequently overexpressed in HCC tissues, where its expression levels strongly correlated with advanced tumor stage and poor prognosis. Functional assays confirmed that UBE2V1 enhances multiple malignant phenotypes of HCC cells. To elucidate the oncogenic mechanism of UBE2V1, we performed proteomic profiling and identified VHL as its primary interacting partner. Specifically, UBE2V1 binds to the β-domain of VHL and, in concert with UBE2S, catalyzes K11- and K48-linked ubiquitination at the K196 residue of VHL, leading to its proteasomal degradation. This event disrupts VHL-mediated ubiquitination and degradation of HIF-1α, thereby enhancing HIF-1α protein stability. Consequently, a positive feedback loop is established between UBE2V1 and HIF-1α, amplifying the expression of key oncogenic downstream effectors such as *VEGF*, *GLUT1*, and *TWIST*. Importantly, therapeutic disruption of the UBE2V1–HIF-1α axis markedly suppressed HCC tumorigenesis and metastasis in vivo, underscoring its potential as a target for novel therapeutic strategies in HCC.

## Results

### The hypoxic tumor microenvironment contributes to the up-regulation of UBE2V1 in HCC

**Fig. 1.**
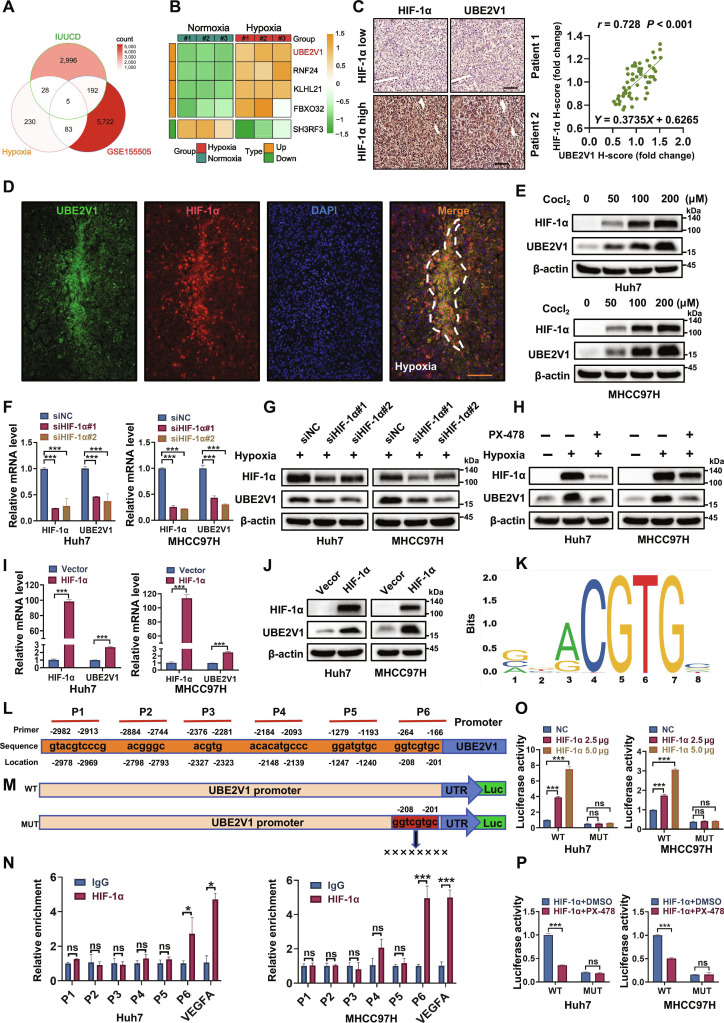
The hypoxic tumor microenvironment contributes to the up-regulation of UBE2V1 in HCC. (A) Venn diagram of ubiquitin-related genes identified based on differential gene expression between normoxia and hypoxia, the public dataset GSE155505, and the human iUUCD database. (B) Heatmap of coexpressed ubiquitin-related genes. (C) The expression levels of HIF-1α and UBE2V1 measured by immunohistochemistry (IHC) at identical tumor sites (left panel), and their correlation was analyzed (right panel) (*n* = 56, Pearson). (D) IF analysis of HIF-1α and UBE2V1 colocalization in hypoxic regions of human HCC tissues (UBE2V1 labeled in green; HIF-1α labeled in red; DAPI labeled in blue; merged signals displayed in yellow). (E) WB analysis of UBE2V1 expression in HCC cells treated with CoCl_2_. (F and G) mRNA and protein levels of UBE2V1 in HCC cells with HIF-1α knockdown under hypoxic conditions (*n* = 3, 1-way ANOVA). (H) Effects of hypoxia and HIF-1α inhibitor on UBE2V1 protein expression levels. (I and J) mRNA and protein levels of UBE2V1 in HCC cells overexpressing HIF-1α (*n* = 3, unpaired Student’s *t* test). (K) HIF-1α binding motif on the *UBE2V1* promoter predicted by the JASPAR database. (L) Schematic diagram of HIF-1α protein binding to the *UBE2V1* promoter motif. (M) Schematic of the wild-type (WT) and mutated promoter (MUT) in the UBE2V1-luciferase reporter gene construct. (N) ChIP-qPCR analysis of HIF-1α protein binding to the *UBE2V1* promoter motif in HCC cells (*n* = 3, unpaired Student’s *t* test). (O) Relative luciferase activity driven by the WT or MUT *UBE2V1* promoter in response to HIF-1α overexpression (*n* = 3, 1-way ANOVA). (P) Relative luciferase activity of the WT or MUT *UBE2V1* promoter under HIF-1α overexpression with or without HIF-1α inhibitor treatment (*n* = 3, unpaired Student’s *t* test). Scale bars: 100 μm. *P < 0.05, ***P < 0.001. ns, not significant.

The hypoxic tumor microenvironment exerts an important influence on the initiation and progression of HCC [20]. To further elucidate this mechanism, we conducted RNA sequencing (RNA-seq) on Huh7 cell lines treated with the hypoxia mimetic cobalt chloride (CoCl_2_). Transcriptomic profiling revealed 2,662 up-regulated and 2,335 down-regulated genes (Fig. S1A). Gene Ontology (GO) functional enrichment and Kyoto Encyclopedia of Genes and Genomes (KEGG) pathway analyses demonstrate a marked correlation between hypoxia and ubiquitination processes (Fig. S1B). Given the crucial role of the ubiquitination pathway in cellular responses to hypoxia [21,22], we aimed to identify key ubiquitin-related hypoxia response factors for further investigation. By overlapping differentially expressed genes under normoxic and hypoxic conditions, data from the Gene Expression Omnibus (GEO) database GSE155505, and the integrated annotations for the integrated Ubiquitination-related Cancer Database (iUUCD), we narrowed down the list to 5 potential candidate genes (Fig. 1A). Comparative analysis of the heatmap revealed *UBE2V1* as the top up-regulated candidate, implying it as a hypoxia-responsive gene with probable mechanistic involvement in hypoxia-driven pathways (Fig. 1B). To prove this, we detected the relationship between hypoxia and UBE2V1 in HCC patient tissues using immunohistochemistry (IHC) and immunofluorescence (IF). The results demonstrated that UBE2V1 was significantly increased in hypoxic HCC tissues and exhibited a pronounced positive correlation with HIF-1α levels (Fig. 1C and D). Moreover, treatment with increasing concentrations of CoCl₂ induced a dose-dependent increase of both UBE2V1 and HIF-1α protein levels (Fig. 1E). Additionally, knocking down HIF-1α significantly reduced the mRNA and protein levels of UBE2V1 under hypoxic conditions (Fig. 1F and G). The same result was observed in PX-478 (HIF-1α inhibitor) treatment (Fig. 1H). Conversely, overexpression of HIF-1α led to a marked increase in both mRNA and protein levels of UBE2V1, further supporting the role of HIF-1α as an upstream regulator of UBE2V1 (Fig. 1I and J). Bioinformatic analysis of the *UBE2V1* promoter (−2,978 to −201 bp) via the JASPAR database predicted 6 putative HREs, designated P1 to P6, which contain the canonical HIF-1α binding core sequence (Fig. 1K and L). Subsequent chromatin immunoprecipitation (ChIP) assay revealed significantly higher enrichment of HIF-1α at the P6 HRE site of the *UBE2V1* promoter compared to the immunoglobulin G (IgG) control group, with VEGFA used as a positive control (Fig. 1N). To further validate this interaction, luciferase reporter vectors containing wild-type (WT) or P6 mutant (MUT) *UBE2V1* promoters were constructed (Fig. 1M). Dual-luciferase reporter assays demonstrated that HIF-1α increased the transcription of *UBE2V1* WT promoter in a dose-dependent manner, whereas no significant change was observed in the MUT group (Fig. 1O). In addition, treatment with the HIF-1α inhibitor reduced luciferase activity in the WT group, but not in the MUT group (Fig. 1P). Collectively, these results demonstrate that the hypoxic tumor microenvironment of HCC induces UBE2V1 up-regulation by promoting HIF-1α binding to the P6 region (−208 to −201 bp, GGTCGTGC) of its promoter.

To assess whether UBE2V1 contributes to the functional role of HIF-1α, we performed reciprocal molecular interventions: either HIF-1α knockdown with UBE2V1 overexpression or HIF-1α overexpression with UBE2V1 knockdown (Fig. S2A and C). Notably, UBE2V1 overexpression partially rescued the growth suppression induced by HIF-1α depletion (Fig. S2B, E, and G), while UBE2V1 knockdown significantly attenuated HIF-1α-driven proliferation as determined by Cell Counting Kit-8 (CCK-8) and colony formation assays (Fig. S2D, F, and H). These results demonstrate that HIF-1α partially executes its pro-tumorigenic functions through UBE2V1.

### High expression of UBE2V1 predicts survival disadvantage in HCC

**Fig. 2.**
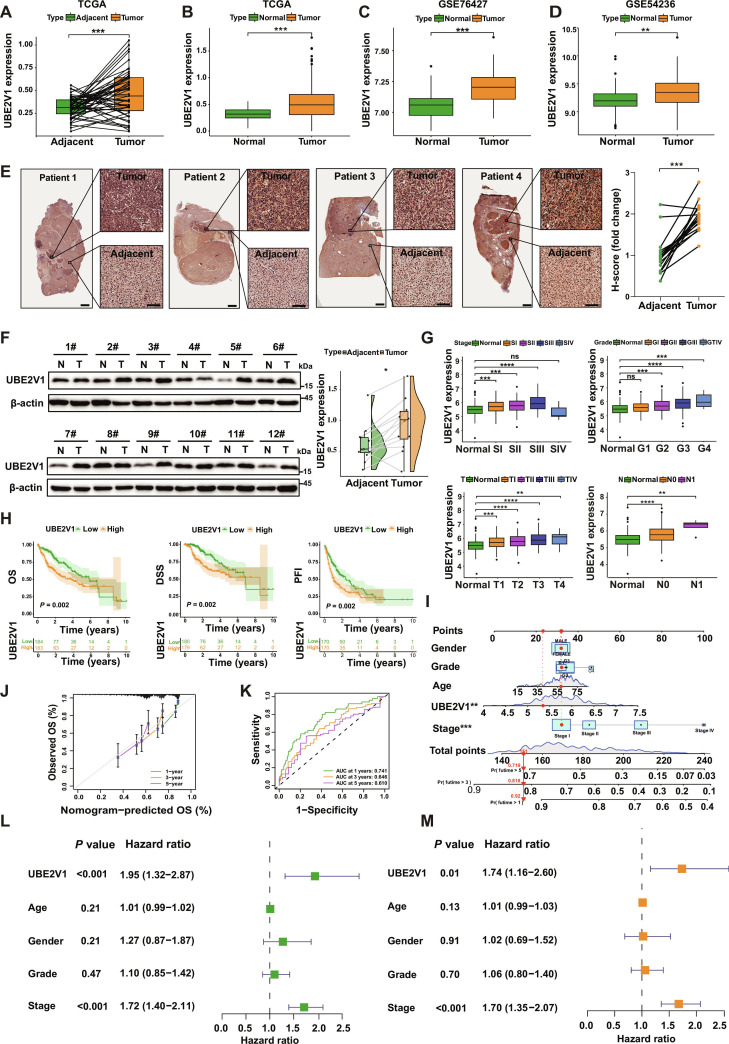
High expression of UBE2V1 predicts survival disadvantage in HCC. (A) Analysis of UBE2V1 mRNA levels in tumors compared with paired adjacent nontumor tissues from TCGA data. (B) UBE2V1 mRNA levels were analyzed between tumors and unpaired normal tissues using TCGA datasets. (C and D) Validation using independent GEO datasets (GSE76427 and GSE54236) of UBE2V1 mRNA levels in tumors versus normal controls. (E) IHC staining of UBE2V1 in tumor and adjacent tissues from representative HCC patients (*n* = 17, paired Student’s *t* test). (F) WB analysis validating UBE2V1 protein expression in tumor and adjacent tissues from patients (*n* = 12, paired Student’s *t* test). (G) The association between UBE2V1 mRNA expression and clinicopathological parameters, including tumor histological stage, differentiation grade, T stage, and N stage. (H) Kaplan–Meier survival curves depicting overall survival (OS), disease-specific survival (DSS), progression-free interval (PFI) in patient groups stratified by high or low UBE2V1 expression using the log-rank test. (I) The nomogram integrates UBE2V1 expression and clinical variables to predict 1-, 3-, and 5-year OS probabilities, along with the relationship between nomogram scores and individual predictors. (J) Calibration curve assessing the agreement between predicted and observed OS probabilities. (K) Receiver operating characteristic (ROC) curve evaluating the predictive accuracy of the UBE2V1 for OS. (L) Univariate Cox regression analysis of potential prognostic factors for OS. (M) Multivariate Cox regression analysis identifying independent prognostic indicators for OS. Scale bars: 1 mm (low magnification) and 100 μm (high magnification). *P < 0.05, **P < 0.01, ***P < 0.001, ****P < 0.0001.

To investigate the expression pattern of UBE2V1 during tumorigenesis, we conducted differential analysis based on the Genotype-Tissue Expression (GTEx), The Cancer Genome Atlas (TCGA) program, and GEO databases. Pan-cancer analysis demonstrated that UBE2V1 was enhanced in the majority of cancer types (Fig. S3A). Specifically, UBE2V1 mRNA levels were significantly elevated in HCC tumor tissues compared to paired adjacent normal controls (Fig. 2A), with box plot validation confirming consistent overexpression across independent cohorts (Fig. 2B to D). More importantly, IHC and Western blot (WB) analyses of paired tumor specimens revealed a significant up-regulation of UBE2V1 protein abundance in HCC tissues compared to adjacent nontumor tissues (Fig. 2E and F). Additionally, both mRNA and protein levels of UBE2V1 were markedly higher in HCC cell lines (LM3, Hep3B, Huh7, HepG2, MHCC97H, and PLC/PRF/5) than in the normal hepatocyte cell line THLE-2 (Fig. S3B and C).

Furthermore, UBE2V1 expression demonstrated significant clinicopathological correlations: UBE2V1 mRNA levels exhibited a progressive increase across TNM stages. Similarly, higher expression levels were observed in tumors with advanced histological grades, more progressed T stages, and lymph node metastases (Fig. 2G). To evaluate the prognostic significance of UBE2V1 expression, we conducted Kaplan–Meier survival analysis and found that patients with high UBE2V1 expression had significantly shorter OS, disease-specific survival (DSS), and progression-free interval (PFI) (Fig. 2H), highlighting a strong correlation between UBE2V1 overexpression and poor clinical outcomes. Based on these findings, we constructed a predictive nomogram integrating UBE2V1 expression with clinical variables (including gender, age, tumor grade, and stage) to estimate 1-, 3-, and 5-year OS probabilities (Fig. 2I). The calibration curve revealed a high degree of agreement between predicted and observed survival probabilities, supporting the clinical applicability of the nomogram in prognostic evaluation (Fig. 2J). Furthermore, ROC curve analysis demonstrated that UBE2V1 exhibited strong discriminatory power in predicting patient survival (Fig. 2K). We further assessed the prognostic value of UBE2V1 using both univariate and multivariate Cox regression analyses. Univariate analysis identified high UBE2V1 expression as a significant risk factor for adverse survival outcomes (Fig. 2L). Multivariate analysis confirmed that UBE2V1 overexpression remained an independent prognostic factor, indicating its potential utility as an independent prognostic biomarker (Fig. 2M).

### UBE2V1 promotes HCC cell proliferation and metastasis

**Fig. 3.**
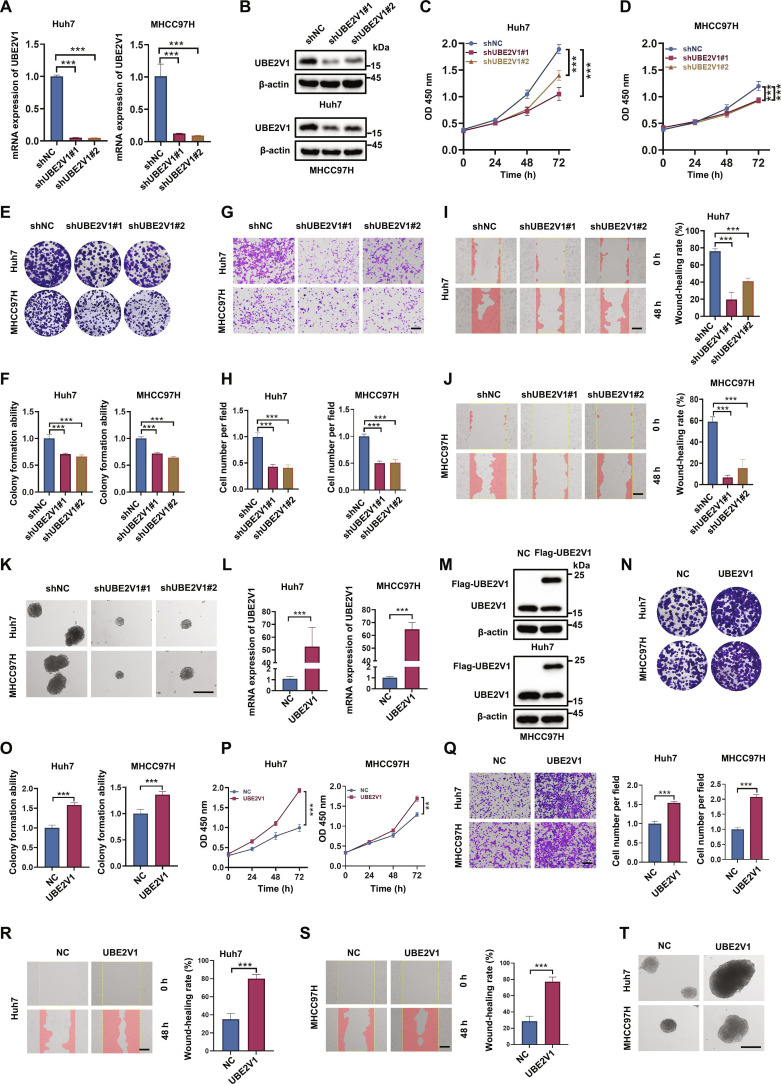
UBE2V1 significantly promotes the malignant behavior of HCC cells. (A and B) Stable UBE2V1-knockdown cell lines were generated in Huh7 and MHCC97H cells using lentiviral transduction. RT-qPCR and WB were performed to confirm the down-regulation of UBE2V1 at both mRNA and protein levels (*n* = 3, 1-way ANOVA). (C and D) CCK-8 and (E and F) colony formation assays demonstrated that depletion of UBE2V1 significantly suppressed the proliferation of Huh7 and MHCC97H cells (*n* = 3, 1-way ANOVA). (G and H) Transwell and (I and J) wound healing experiments indicated that UBE2V1 knockdown markedly reduced the migratory potential of these cells (*n* = 3, 1-way ANOVA). (K) Spheroid formation assays revealed a decrease in the tumor sphere-forming ability of HCC cells following UBE2V1 silencing. (L and M) Stable UBE2V1-overexpressing Huh7 and MHCC97H cell lines were established using lentiviral infection (*n* = 3, unpaired Student’s *t* test). RT-qPCR and WB were used to determine the mRNA and protein levels of UBE2V1. (N and O) Subsequent colony formation and (P) CCK-8 assays demonstrated that UBE2V1 overexpression significantly enhanced cell proliferation (*n* = 3, unpaired Student’s *t* test). (Q) Transwell assays and (R and S) wound healing further revealed that UBE2V1 up-regulation promoted cell migration (*n* = 3, unpaired Student’s *t* test). (T) Spheroid formation capacity was increased upon UBE2V1 overexpression. Scale bars: 200 μm. **P < 0.01, ***P < 0.001.

To explore the functional role of UBE2V1 in regulating the malignant phenotype, we established stable HCC cell lines with either knockdown or overexpression of UBE2V1. Real-time quantitative polymerase chain reaction (RT-qPCR) and WB analyses confirmed that 2 distinct short hairpin RNA (shRNA) constructs effectively down-regulated UBE2V1 expression in both Huh7 and MHCC97H cell lines (Fig. 3A and B). CCK-8 and colony formation assays demonstrated that UBE2V1 knockdown significantly inhibited the proliferation of HCC cells (Fig. 3C to F). Moreover, transwell migration (Fig. 3G and H) and wound healing assays (Fig. 3I and J) revealed that UBE2V1 depletion dramatically impaired the migratory capacity of HCC cells. Assessment of stem-like properties showed a marked reduction in tumor sphere formation in UBE2V1 knockdown cells (Fig. 3K). By contrast, UBE2V1 overexpression exhibited an opposite effect on HCC proliferation and metastasis. The efficiency of UBE2V1 overexpression is presented in Fig. 3L and M. Functional assays revealed that UBE2V1 overexpression significantly enhanced cell growth (Fig. 3N to P), migratory activity (Fig. 3Q to S), and tumor sphere formation capacity (Fig. 3T). Collectively, these findings demonstrate that elevated UBE2V1 expression promotes tumor cell proliferation and metastasis, which aligns with clinical observations linking high UBE2V1 levels to poor patient prognosis, suggesting its importance as an oncogenic driver and a potential therapeutic target in HCC progression.

### UBE2V1 interacts with VHL

**Fig. 4.**
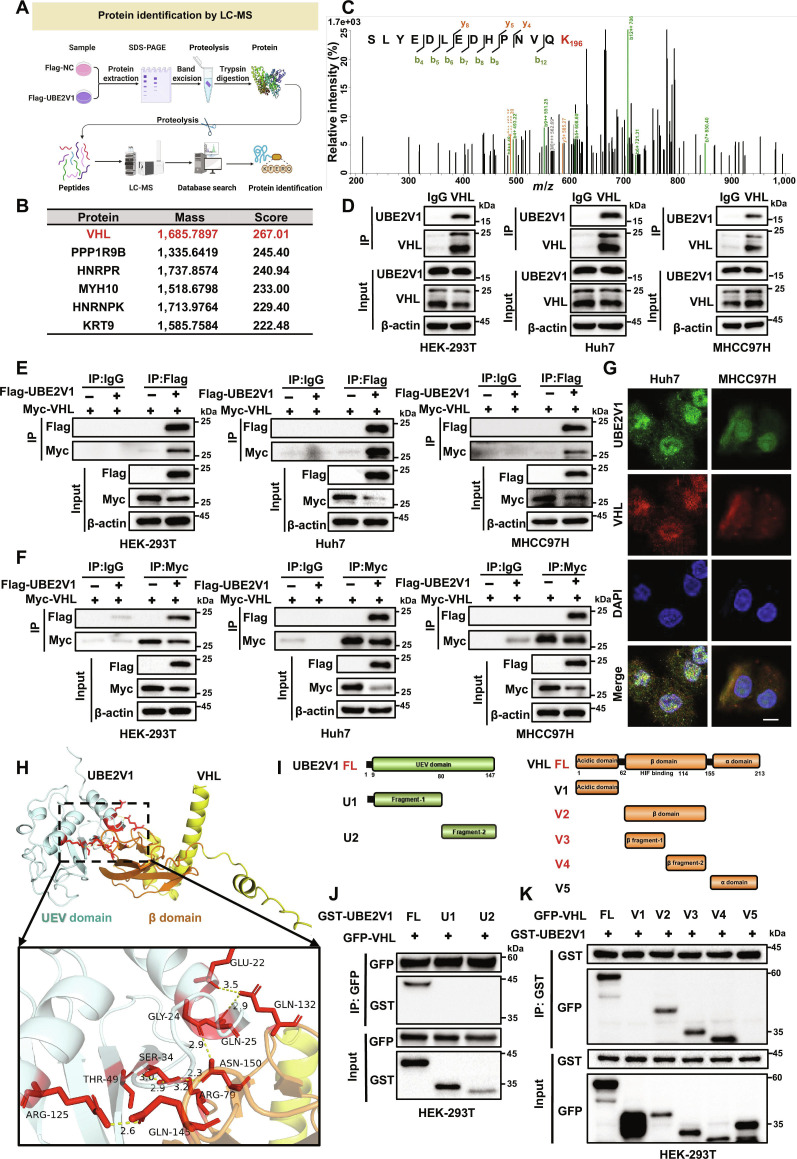
UBE2V1 interacts with VHL. (A) Schematic diagram of the LC-MS/MS workflow for analyzing the Flag-UBE2V1 IP complex. (B) Top 6 enriched proteins were identified in the IP samples. (C) Tandem mass spectrum of the VHL peptide segment. (D) Co-IP validation of the endogenous interaction between UBE2V1 and VHL. (E) Co-IP assays demonstrated the exogenous interaction between Flag-UBE2V1 and Myc-VHL using a Flag antibody. (F) Co-IP results confirming the binding between Flag-UBE2V1 and Myc-VHL with a Myc antibody. (G) IF colocalization analysis demonstrated the intracellular colocalization of UBE2V1 and VHL. UBE2V1 (green fluorescence), VHL (red fluorescence), and nuclei (blue fluorescence) were observed, with yellow regions indicating colocalization. (H) Molecular docking model illustrating the interaction between UBE2V1 and VHL (ipTM = 0.2, pTM = 0.48). The cyan structure represents UBE2V1, the yellow structure denotes the VHL protein, the red highlighted region indicates the critical interaction interface, and dashed lines represent hydrogen bonds. (I) Schematic representation of the full-length and truncation mutants of UBE2V1 and VHL. (J) Mapping the interaction of UBE2V1 constructs with VHL. (K) Mapping the interaction of VHL constructs with UBE2V1. Scale bars: 10 μm.

To elucidate the oncogenic mechanisms driven by UBE2V1 in HCC, we performed proteomic profiling to identify potential interacting proteins of UBE2V1. Following IP of Flag-UBE2V1, mass spectrometry (MS) analysis identified VHL as the predominant interactor with the highest peptide score of 267.01 (Fig. 4A and B). Tandem MS analysis confirmed the presence of the unique VHL peptide SLYEDLEDHPNVQK (Fig. 4C) along with 2 UBE2V1 peptides (Fig. S4). In HEK-293T cells, endogenous VHL effectively pulled down UBE2V1, and this interaction was further confirmed in HCC cell lines under endogenous conditions (Fig. 4D). Forward co-immunoprecipitation (Co-IP) assays demonstrated that Flag-UBE2V1 specifically binds to Myc-VHL (Fig. 4E). Reverse IP experiments confirmed that Myc-VHL also interacts with Flag-UBE2V1 (Fig. 4F), with consistent results observed in 3 distinct cell lines. IF colocalization analysis revealed that UBE2V1 (green fluorescence) and VHL (red fluorescence) colocalize in both the cytoplasm and nucleus (Fig. 4G). Additionally, molecular docking analysis of the UBE2V1–VHL complex provided structural insights into their interaction. The model indicates that UBE2V1 (cyan) binds to the α-helical groove of VHL (yellow) via its β-sheet domain, forming a complementary interaction interface (highlighted in red) (Fig. 4H). This detailed view of the binding site reveals a network of polar interactions, providing structural evidence for the direct physical association between UBE2V1 and VHL. Previous studies have clearly defined the structural domains of VHL [23,24]. To identify the specific domains mediating the interaction between UBE2V1 and VHL, we generated 2 GST-UBE2V1 and 5 GFP-VHL truncated mutants (Fig. 4I). After transfection into HEK-293T cells and subsequent Co-IP assays, we found that VHL specifically interacts with full-length UBE2V1 but not with its individual N- or C-terminal truncated variants (Fig. 4J). Meanwhile, the β-domain of VHL (amino acids 62 to 155) was identified as critical for UBE2V1 interaction (Fig. 4K). Collectively, these data establish a direct UBE2V1–VHL interaction in HCC cells and suggest its functional relevance in governing tumor progression.

### UBE2V1 targets VHL for ubiquitin-mediated degradation to promote HCC progression

**Fig. 5.**
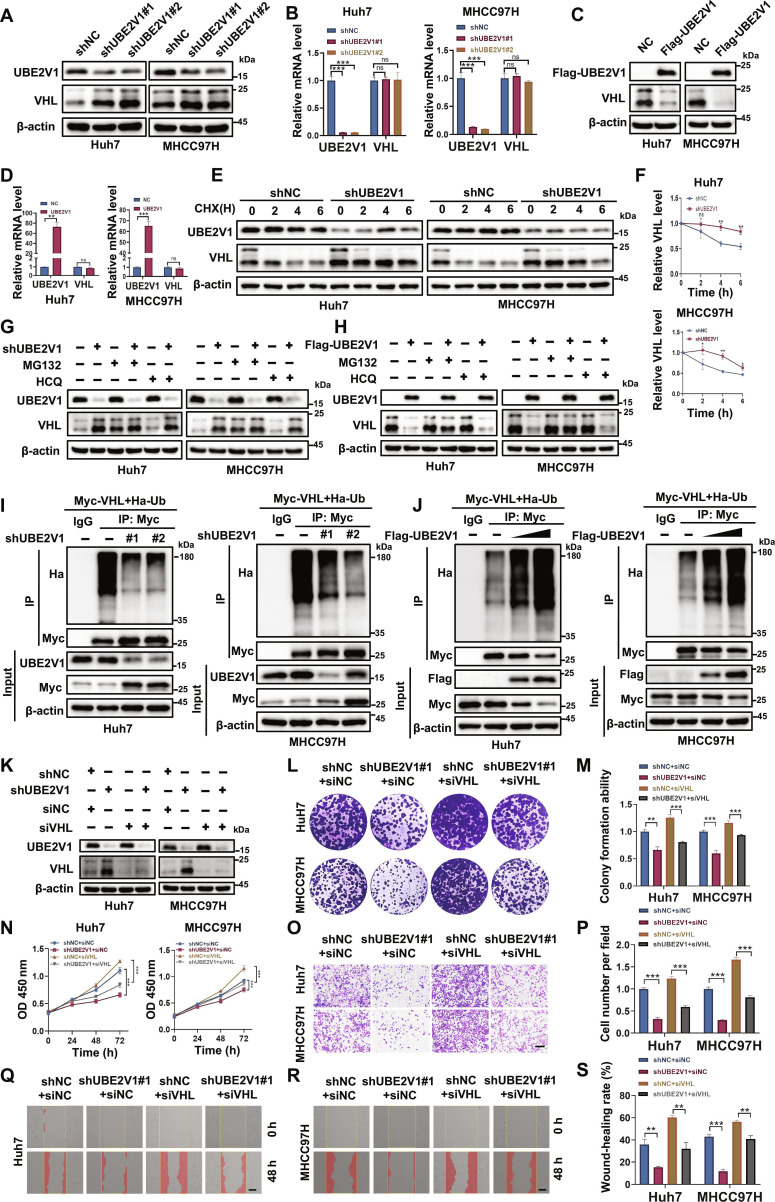
UBE2V1 targets VHL for ubiquitin-mediated degradation to promote HCC progression. (A and B) Effect of UBE2V1 knockdown on VHL protein and mRNA levels (*n* = 3, 1-way ANOVA). (C and D) Effect of UBE2V1 overexpression on VHL protein and mRNA levels (*n* = 3, unpaired Student’s *t* test). (E and F) CHX assays were performed to determine the half-life of VHL in Huh7 cells (*n* = 3, 2-way ANOVA) and MHCC97H cells (*n* = 3, 2-way ANOVA). (G and H) Protein levels were measured in HCC cells treated with MG132 or HCQ. (I) Effect of UBE2V1 knockdown on VHL ubiquitination levels. (J) Effect of UBE2V1 overexpression on VHL ubiquitination levels. (K) WB analysis of protein expression levels in HCC cells with UBE2V1 knockdown and siVHL. (L and M) Colony formation assay and (N) CCK-8 assay to evaluate cell proliferation capacity (*n* = 3, 1-way ANOVA). (O and P) Transwell assay and (Q to S) wound healing assay to assess cell migration ability (*n* = 3, 1-way ANOVA). Scale bars: 200 μm. *P < 0.05, **P < 0.01, ***P < 0.001. ns, not significant.

The interaction between E2 ubiquitin-conjugating enzymes UBE2V1 and VHL led us to investigate its impact on VHL protein levels. Following the knockdown of UBE2V1, a marked increase in VHL protein levels was detected in both Huh7 and MHCC97H cell lines (Fig. 5A). Notably, RT-qPCR analysis showed that silencing UBE2V1 did not affect VHL mRNA levels (Fig. 5B). Moreover, overexpression of UBE2V1 decreased VHL protein levels without altering its mRNA levels (Fig. 5C and D), suggesting that UBE2V1 regulates VHL through a posttranscriptional mechanism. To assess the role of UBE2V1 in modulating VHL stability, cycloheximide (CHX) was used to inhibit novel protein synthesis. As seen in Fig. 5E and F, depletion of UBE2V1 significantly prolonged the half-life of VHL in Huh7 and MHCC97H cells. Furthermore, the involvement of the proteasomal pathway in UBE2V1-mediated VHL degradation was confirmed by the fact that the proteasome inhibitor MG132 effectively reversed this process, whereas the lysosome inhibitor hydroxychloroquine (HCQ) had no effect (Fig. 5G and H). We then examined whether UBE2V1 promotes VHL ubiquitination. As anticipated, UBE2V1 silencing dramatically reduced VHL ubiquitination levels (Fig. 5I), whereas its overexpression enhanced polyubiquitination of VHL (Fig. 5J), indicating that UBE2V1 facilitates VHL ubiquitination and subsequent proteasomal degradation.

Next, to elucidate whether UBE2V1 promotes HCC cell proliferation and metastasis by inhibiting VHL, we transfected VHL small interfering RNA (siRNA) into shNC or UBE2V1-silenced Huh7 and MHCC97H cells (Fig. 5K). As shown in Fig. 5L to S, knockdown of VHL partially rescued the suppressive effects on HCC cell proliferation and migration caused by depletion of UBE2V1, indicating that the oncogenic effects of UBE2V1 are primarily mediated through VHL suppression. In aggregate, UBE2V1 promotes the ubiquitin-mediated proteasomal degradation of VHL, thereby relieving the inhibitory effects of VHL on downstream oncogenic signaling pathways and ultimately facilitating the malignant progression of HCC cells.

### UBE2V1 promotes VHL degradation via K11- and K48-linked polyubiquitination at Lys196 in complex with UBE2S

**Fig. 6.**
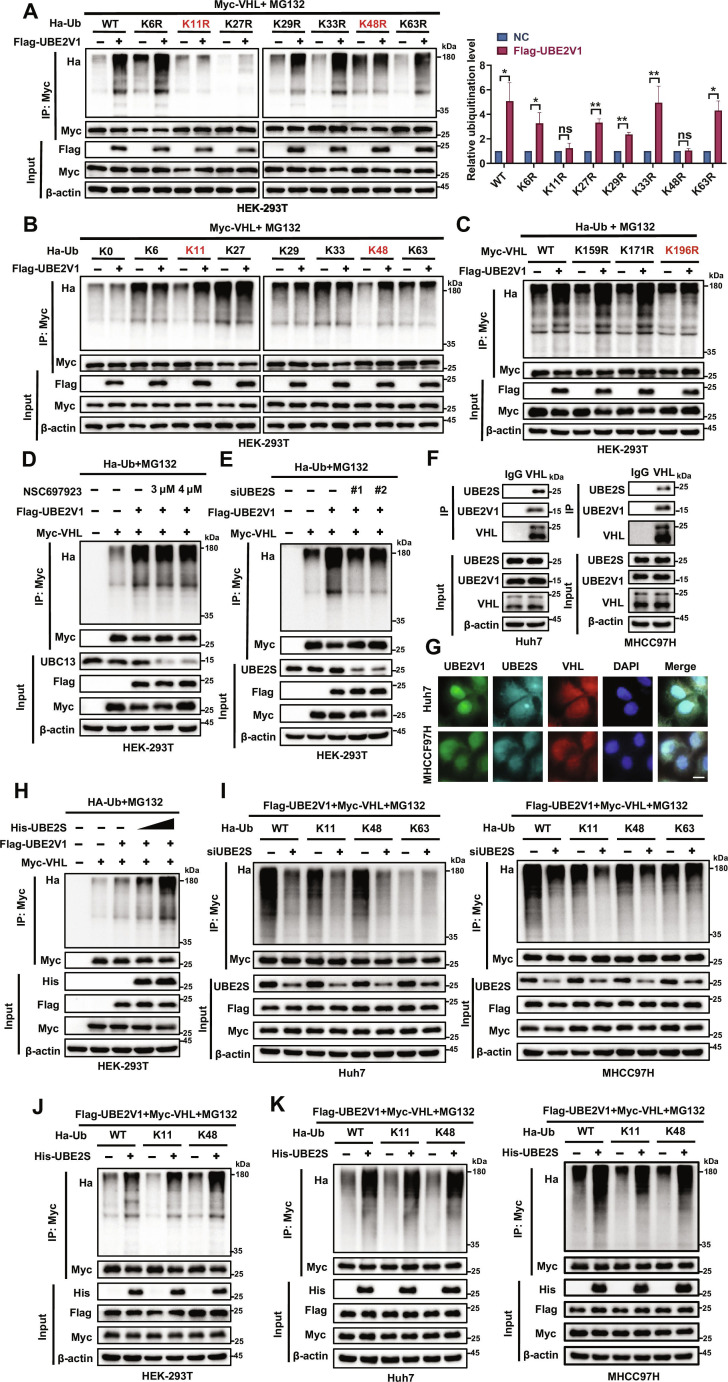
UBE2V1 promotes K11- and K48-linked polyubiquitination of VHL at Lys196 in complex with UBE2S. (A) Analysis of ubiquitin chain types regulated by UBE2V1 on VHL. HEK-293T cells were cotransfected with Flag-UBE2V1, Myc-VHL, and WT or single-site mutant (K6R/K11R/K27R/K29R/K33R/K48R/K63R) HA-Ub plasmids. Ubiquitination of VHL was subsequently analyzed by WB (*n* = 3, unpaired Student’s *t* test). (B) Verification of UBE2V1-mediated ubiquitin chain linkage types on VHL. HEK-293T cells were cotransfected with Flag-UBE2V1, Myc-VHL, and lysine-deficient (K0) or single-lysine-retained (K6/K11/K27/K29/K33/K48/K63) HA-Ub plasmids. Immunoprecipitation and WB were subsequently performed to evaluate VHL ubiquitination. (C) Evaluation of VHL single-site mutations on UBE2V1-mediated ubiquitination. (D) Assessment of the effect of UBC13 inhibition on VHL ubiquitination. (E) Investigation of the impact of UBE2S knockdown on VHL ubiquitination. (F) Confirmation of the interaction among VHL, UBE2V1, and UBE2S in HCC cells. (G) Colocalization among UBE2V1, UBE2S, and VHL. (H) Evaluation of UBE2S overexpression on VHL ubiquitination levels. (I) Specific regulation of VHL ubiquitin chain types by UBE2S knockdown. (J and K) Specific regulation of VHL ubiquitin chain types by UBE2S overexpression. Scale bars: 10 μm. *P < 0.05, **P < 0.01. ns, not significant.

To further delineate the underlying mechanism of UBE2V1-mediated VHL ubiquitination, we first characterized the specific polyubiquitin chain linkages on VHL using in vivo ubiquitination assays in HEK-293T cells. While UBE2V1 overexpression significantly enhanced VHL polyubiquitination in cells expressing Ub-WT or most other lysine-to-arginine mutants, this enhancement was substantially abolished in cells expressing the Ub-K11R or Ub-K48R mutants (Fig. 6A). Consistently, overexpression of UBE2V1 specifically enhanced the binding of VHL to K11- and K48-linked ubiquitin chains (Fig. 6B). These results confirm that UBE2V1-mediated VHL ubiquitination primarily depends on K11- and K48-linkage types, consistent with canonical proteasomal targeting signals in which K48-linked ubiquitin serves as the principal degradation signal, and K11 chains may enhance degradation efficiency through synergistic effects [25–27].

Furthermore, we employed the iUUCD database for sequence analysis, which predicted 3 potential ubiquitination sites on the VHL protein: K159 (ANITLPVYTLKERCLOVVRSL), K171 (RCLQVVRSLVKPENYRRLDIV), and K196 (EDLEDHPNVQKDLERLTQERI) (Fig. S5A). Mutation of all 3 lysine residues to arginine (VHL 3KR) abolished UBE2V1-mediated ubiquitination of VHL (Fig. S5B). Subsequent site-directed mutagenesis experiments revealed that the K196R mutation substantially impaired UBE2V1-induced VHL ubiquitination, while K159R and K171R mutations had no marked effect (Fig. 6C). These results are consistent with our prior MS data that identified ubiquitination at K196 of VHL (Fig. 4C), confirming K196 on VHL as the primary site for UBE2V1-mediated ubiquitination.

As UBE2V1 lacks the conserved catalytic cysteine residue required for E2 enzyme activity, it typically functions by forming a heterodimer with UBC13 [28]. Surprisingly, treatment with the UBC13 inhibitor NSC697923 did not affect the VHL ubiquitination, suggesting that UBE2V1 promotes VHL ubiquitination independently of UBC13 (Fig. 6D). Further investigation revealed that silencing UBE2S, an E2 enzyme recently shown to ubiquitinate and degrade VHL protein [29], markedly diminished UBE2V1-induced polyubiquitination of VHL (Fig. 6E). Subsequent Co-IP assays confirmed a specific interaction between Flag-UBE2V1 and His-UBE2S (Fig. S5C). Endogenous IP further demonstrated that VHL interacts with both UBE2V1 and UBE2S (Fig. 6F). We also observed colocalization among UBE2V1, UBE2S, and VHL in both Huh7 and MHCC97H cells (Fig. 6G). Moreover, exogenous complementation with His-UBE2S increased UBE2V1-mediated VHL ubiquitination (Fig. 6H). Given that Zhang et al. [29] have demonstrated that UBE2S promotes VHL degradation via K11-linked polyubiquitination, we examined whether UBE2S specifically contributes to UBE2V1-mediated K11-linked chain formation. As seen in Fig. 6I and Fig. S5D, knockdown of UBE2S reduced UBE2V1-dependent K11- and K48-linked VHL ubiquitination levels, but not K63-linked chains. In contrast, UBE2S overexpression enhanced UBE2V1-mediated K11 and K48 ubiquitination of VHL (Fig. 6J and K). These findings indicate that UBE2S is essential for UBE2V1 to catalyze K11-/K48-linked polyubiquitination of VHL, likely by forming a functional heterodimeric complex that confers specificity for these chain linkages. In summary, UBE2V1 drives the ubiquitination and degradation of VHL by specifically targeting its K196 site and facilitating K11-/K48-linked chain assembly through the formation of a functional heterodimer with UBE2S.

### UBE2V1 activates HIF-1α by inhibiting VHL-mediated degradation

**Fig. 7.**
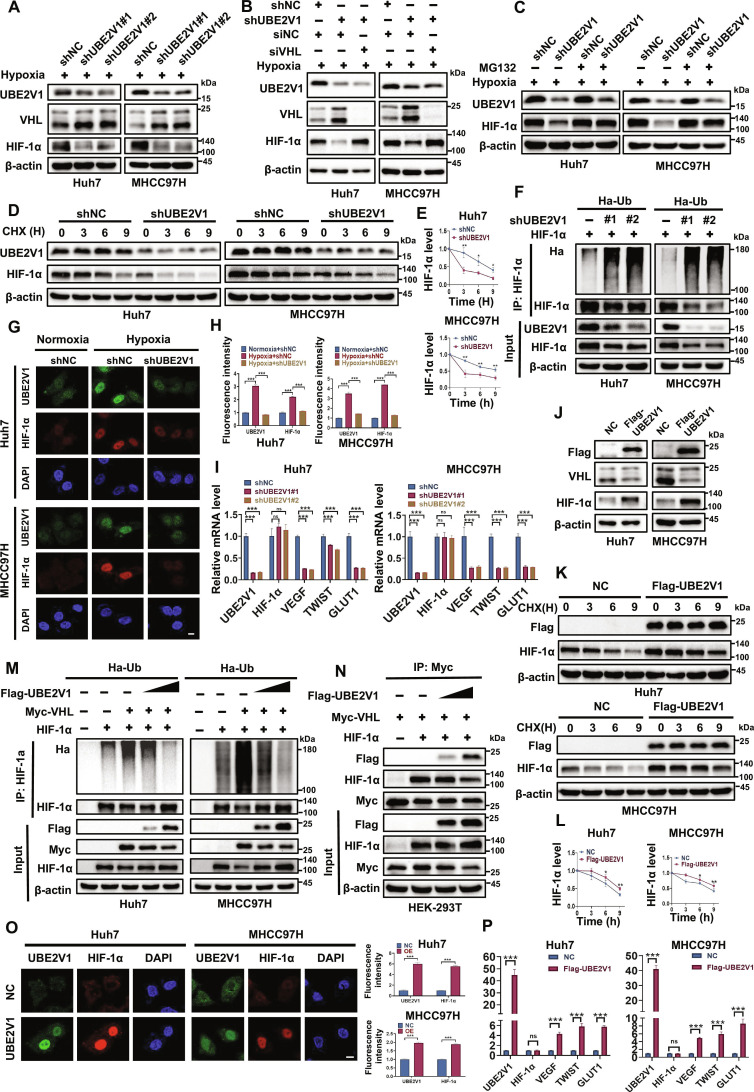
UBE2V1 activates HIF-1α by inhibiting VHL-mediated ubiquitination and degradation of HIF-1α. (A) Effect of UBE2V1 knockdown on VHL and HIF-1α protein levels. (B) Rescue of HIF-1α expression by siVHL in UBE2V1-deficient cells. (C) Proteasome inhibition stabilizes HIF-1α in UBE2V1-knockdown cells. (D and E) Effect of UBE2V1 knockdown on HIF-1α stability (*n* = 3, 2-way ANOVA). (F) UBE2V1 knockdown promotes HIF-1α ubiquitination. (G and H) HIF-1α nuclear localization under normoxia, hypoxia, and hypoxia with UBE2V1 knockdown (UBE2V1: green; HIF-1α: red; DAPI: blue) (*n* = 3, 1-way ANOVA). (I) UBE2V1 knockdown decreases mRNA levels of canonical HIF-1α target genes under hypoxia (*n* = 3, 1-way ANOVA). (J) Effect of UBE2V1 overexpression on VHL and HIF-1α expression. (K and L) UBE2V1 overexpression stabilizes HIF-1α protein (*n* = 3, 2-way ANOVA). (M) UBE2V1 overexpression inhibits HIF-1α ubiquitination. (N) UBE2V1 competes with HIF-1α for VHL binding. (O) UBE2V1 overexpression promotes HIF-1α nuclear accumulation. (UBE2V1: green; HIF-1α: red; DAPI: blue) (*n* = 3, unpaired Student’s *t* test). (P) UBE2V1 overexpression activates canonical HIF-1α target genes (*n* = 3, unpaired Student’s *t* test). Scale bars: 10 μm. *P < 0.05, **P < 0.01, ***P < 0.001. ns, not significant.

As the substrate recognition subunit of the E3 ubiquitin ligase complex, VHL primarily functions to target HIF-1α for proteasomal degradation [30,31]. Given our findings above that UBE2V1 mediates ubiquitin-dependent degradation of VHL, we sought to determine whether UBE2V1 contributes to the regulation of HIF-1α stability. Under hypoxic conditions, UBE2V1 knockdown in Huh7 and MHCC97H cells increased VHL and concurrently decreased HIF-1α protein levels (Fig. 7A), while this effect on HIF-1α was effectively rescued by VHL silencing (Fig. 7B). Treatment with the proteasome inhibitor MG132 effectively blocked HIF-1α degradation induced by UBE2V1 deletion (Fig. 7C). Consistently, CHX chase experiments revealed that UBE2V1 deletion accelerated HIF-1α degradation in both cell lines under hypoxia (Fig. 7D and E). Ubiquitination assays further confirmed that UBE2V1 knockdown substantially enhanced HIF-1α polyubiquitination (Fig. 7F). Moreover, IF analysis indicated elevated nuclear accumulation of both UBE2V1 and HIF-1α under hypoxic versus normoxic conditions. However, UBE2V1 knockdown dramatically reduced HIF-1α fluorescence intensity in nucleus, implying that UBE2V1 not only stabilizes HIF-1α but may also facilitate its nuclear translocation (Fig. 7G and H). Consistent with this, RT-qPCR analysis showed that UBE2V1 depletion significantly reduced the mRNA levels of canonical HIF-1α target genes (*VEGF*, *TWIST*, and *GLUT1*) under hypoxia, while HIF-1α mRNA levels remained unchanged (Fig. 7I).

Conversely, UBE2V1 overexpression led to decreased VHL protein levels and a concomitant increase in HIF-1α expression (Fig. 7J). CHX experiments further confirmed that UBE2V1 overexpression enhanced HIF-1α stability in both Huh7 and MHCC97H cells (Fig. 7K and L). Importantly, UBE2V1 overexpression markedly suppressed VHL-mediated ubiquitination of HIF-1α in a dose-dependent manner (Fig. 7M). Given that the β-domain of VHL serves as the binding site for both HIF-1α [32] and UBE2V1 (Fig. 4K), we investigated whether UBE2V1 competitively binds VHL and disrupts its interaction with HIF-1α. Co-IP assays showed that the binding between VHL and HIF-1α decreased with increasing concentrations of UBE2V1, indicating competitive inhibition and subsequent release of HIF-1α (Fig. 7N). IF analysis also demonstrated that UBE2V1 overexpression significantly promoted HIF-1α nuclear accumulation (Fig. 7O). RT-qPCR analysis demonstrated that UBE2V1 overexpression up-regulated the mRNA levels of HIF-1α target genes, indicating that nuclear HIF-1α exerts transcriptional activity to activate downstream target genes involved in HCC progression (Fig. 7P). In summary, UBE2V1 not only promotes the ubiquitination and degradation of VHL but also competes with HIF-1α for binding to VHL, thereby inhibiting VHL-mediated ubiquitination and degradation of HIF-1α and ultimately leading to its stabilization, nucleus accumulation, and subsequent activation of downstream target genes. These findings establish a closed-loop regulatory axis of “UBE2V1–VHL–HIF-1α” and reveal a novel regulatory mechanism by which HCC tumor cells adapt to the hypoxic microenvironment, offering experimental support for targeting UBE2V1 in the treatment of HCC.

### HIF-1α is required for UBE2V1 to promote HCC progression both in vitro and in vivo

**Fig. 8.**
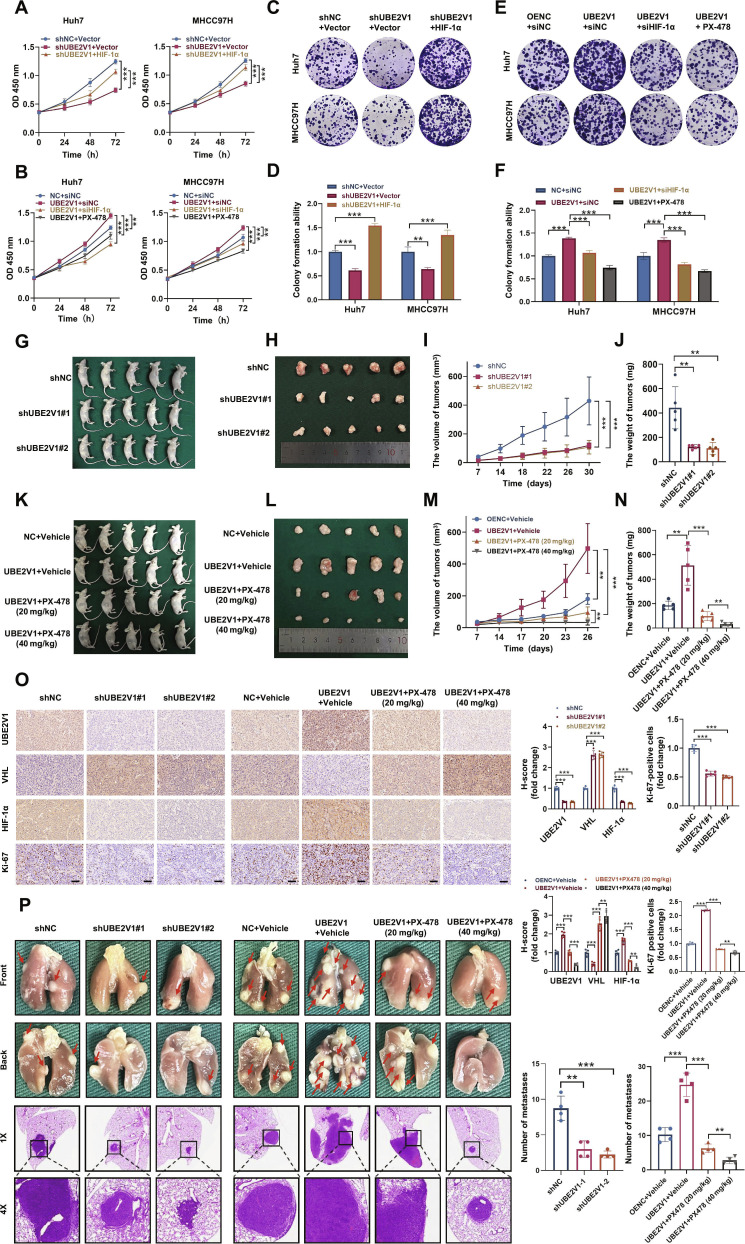
HIF-1α is required for UBE2V1 to promote HCC progression both in vitro and in vivo. (A and B) CCK-8 assay for evaluating cellular proliferation capacity (*n* = 3, 1-way ANOVA). (C to F) Colony formation assay demonstrating differential cellular proliferation (*n* = 3, 1-way ANOVA). (G and K) Euthanasia and photographic documentation of tumor-bearing nude mice. (H and L) Tumor imaging in nude mice. (I and M) Tumor volume was measured over the indicated time course (*n* = 5, 2-way ANOVA). (J and N) Final tumor weight was measured at the endpoint (*n* = 5, 1-way ANOVA). (O) Representative IHC images of tumor sections stained for UBE2V1, VHL, HIF-1α, and Ki-67 (*n* = 5, 1-way ANOVA). (P) Intravenous injection model (*n* = 4, 1-way ANOVA). Scale bars: 50 μm. **P < 0.01, ***P < 0.001.

Having established that UBE2V1 stabilizes and activates HIF-1α, we next investigated whether HIF-1α plays a critical role in mediating the oncogenic functions of UBE2V1 in HCC. In Huh7 and MHCC97H cells, knockdown of UBE2V1 significantly impaired cellular proliferation, an effect that was reversed by transfection with a HIF-1α expression vector (Fig. 8A, C, and D). Conversely, overexpression of UBE2V1 markedly enhanced cell growth, which was abolished upon transfection with siHIF-1α or HIF-1α-specific inhibitor (Fig. 8B, E, and F). To validate whether UBE2V1 exerts its tumor-promoting effects in vivo and to assess the involvement of HIF-1α activation in this process, MHCC97H cells stably expressing shUBE2V1 or shNC were subcutaneously injected into nude mice (Fig. S6A). The subcutaneous tumorigenesis assay demonstrated that UBE2V1 knockdown markedly inhibited tumor growth, with marked reductions in both tumor volume and weight. (Fig. 8G to J). In contrast, UBE2V1 overexpression significantly increased tumor growth, while systemic administration of PX-478 suppressed tumor development in a dose-dependent manner, confirming that the pro-tumorigenic effects of UBE2V1 in HCC are mediated through HIF-1α activation (Fig. 8K to N). IHC analysis confirmed elevated VHL expression, reduced HIF-1α levels, and a lower Ki-67 proliferation index in tumors derived from UBE2V1 knockdown cells. By comparison, tumors overexpressing UBE2V1 exhibited decreased VHL expression, increased HIF-1α levels, and enhanced Ki-67 staining, whereas PX-478 treatment effectively rescued these effects (Fig. 8O). The relevant H&E staining results are presented in Fig. S6B. WB analysis further demonstrated up-regulation of VHL in the shUBE2V1 group, while down-regulation of VHL was observed in the UBE2V1 group (Fig. S6C and D). Tail vein injection experiments further showed that UBE2V1 deletion significantly reduced lung metastasis, whereas UBE2V1 overexpression enhanced the number of metastatic foci compared to control groups. Importantly, combined treatment with PX-478 significantly attenuated metastatic spread in the UBE2V1-overexpressing group (Fig. 8P). Taken together, these results demonstrate the pivotal role of HIF-1α in UBE2V1-driven HCC progression in vitro and in vivo.

## Discussion

HCC constitutes approximately 90% of all primary liver cancers and remains a highly aggressive malignancy associated with high mortality and poor global prognosis [1,33]. Despite significant progress in localized, targeted, and immunotherapeutic approaches, most HCC patients still face unfavorable clinical outcomes and ultimately succumb to the disease [34–36]. A key factor underlying therapeutic failure is the highly hypoxic tumor microenvironment, a hallmark of HCC characterized by a median intratumoral oxygen level as low as 6 mmHg, compared to 30 mmHg in normal liver tissue [37–39]. Hypoxia and HCC progression form a bidirectional vicious cycle: preexisting liver pathologies induce microcirculatory dysfunction and chronic hypoxia, which activates HIF signaling and promotes a procarcinogenic microenvironment; subsequently, rapid tumor growth and aberrant vascularization further intensify hypoxia, creating a self-reinforcing loop [40–42]. Within this hypoxic circuit, we identified *UBE2V1* as a key hypoxia-responsive gene that is transcriptionally activated by HIF-1α through direct binding to the HRE in the *UBE2V1* promoter region. These findings expand the canonical hypoxia signaling network by establishing *UBE2V1* as a novel transcriptional target of the hypoxia–HIF-1α axis.

Previous studies have implicated UBE2V1 as a potential oncoprotein across multiple cancer types. For instance, UBE2V1-mediated ubiquitination and degradation of Sirt1 promote colorectal cancer metastasis through epigenetic suppression of autophagy [17]. In melanoma, the interaction between UBE2V1 and UBC13 promotes tumor growth via the MEK/FRA1/SOX10 signaling pathway [28]. Additionally, UBE2V1 promotes osteosarcoma differentiation through Smurf1-dependent ubiquitination and subsequent degradation of Smad1 [43]. However, the functional significance of the UBE2V1 in HCC remains poorly characterized. In this study, analyses of TCGA and GEO datasets, along with validation in clinical specimens, revealed significant up-regulation of UBE2V1 in HCC tissues, which correlates with advanced tumor stage, tumor grade, and lymph node metastasis. Multivariate Cox regression analysis confirmed UBE2V1 as an independent prognostic factor, highlighting its potential as a prognostic biomarker for HCC. A nomogram integrating UBE2V1 expression with clinical variables demonstrated robust predictive accuracy, offering a practical tool for risk stratification. Furthermore, loss-of-function and gain-of-function experiments clearly demonstrated that UBE2V1 promotes HCC cell proliferation and migration. These effects are largely dependent on HIF-1α, as HIF-1α knockdown or treatment with the inhibitor PX-478 abolished UBE2V1-driven oncogenic phenotypes. Consistent with this, UBE2V1 overexpression activated key HIF-1α downstream targets including *VEGF*, *GLUT1*, and *TWIST*. Interestingly, UBE2V1 also modulates the expression of c-Myc and LIN28B (Fig. S7A and B). c-Myc is a well-known proto-oncoprotein frequently activated in cancer and associated with altered metabolism, stemness, and aggressive tumor behavior [44–46]. LIN28B, on the other hand, promotes stem cell self-renewal and tumor metastasis by inhibiting members of the let-7 miRNA family [47–49]. The coordinated regulation of c-Myc and LIN28B by UBE2V1 suggests potential HIF-1α-independent mechanisms contributing to HCC progression, warranting further investigation.

The VHL protein, a tumor suppressor and core component of an E3 ubiquitin ligase complex, directly interacts with the oxygen-dependent degradation domain of HIF-1α (amino acids 401 to 603) via residues such as Tyr^98^, His^115^, and Ser^111^ in its β-domain, recruiting E2 enzymes and mediating HIF-1α ubiquitination and proteasomal degradation [50]. Mutations in the VHL β-domain (Y98H and S111L) disrupt this interaction, thereby preventing HIF-1α degradation [32]. In many cancers, VHL gene mutations or deletions result in loss of VHL function, leading to HIF-1α accumulation and tumor progression [51–53]. Notably, our findings indicate that UBE2V1 expression was elevated in HCC, resulting in continuous suppression of VHL function and impairing its ability to target HIF-1α for degradation. This effect phenocopies the loss of VHL function observed in VHL-mutated cancers, a conclusion further supported by our functional rescue experiments showing that knockdown of VHL counteracts the reduction in oncogenic behaviors resulting from UBE2V1 knockdown in HCC cells. Interestingly, we observed that UBE2V1 knockdown enhanced VHL protein levels, leading to reduced HIF-1α protein levels even under hypoxic conditions (Fig. 7A and B). While this finding may appear to differ from the classical understanding, several lines of evidence may help explain this phenomenon. Firstly, under moderate hypoxia, prolyl hydroxylase activity is partially maintained, allowing a proportion of HIF-1α molecules to undergo hydroxylation and subsequent recognition by VHL, as evidenced by detectable hydroxylated HIF-1α in hypoxic tumor regions and moderately hypoxic cells [54,55]. Secondly, the key oxygen sensor PHD2 is itself transcriptionally induced by HIF-1α, establishing a feedback loop that makes the degradation process highly dynamic [56,57]. Importantly, previous studies have shown that VHL can interact with and promote degradation of SUMOylated HIF-1α through hydroxylation-independent mechanisms during hypoxia [58]. Collectively, these studies provide a plausible explanation for how elevated VHL levels enhance HIF-1α degradation even in hypoxic conditions.

As a catalytically inactive E2 variant, UBE2V1 requires heterodimerization with functional E2 enzymes to exert its biological effects, and UBC13 has been identified as a primary interacting partner. For instance, in colorectal cancer, UBE2V1 forms heterodimers with UBC13, where UBC13 provides catalytic activity while UBE2V1 enhances affinity with E3 ligases such as TRAF6, promoting K63-linked ubiquitin chain synthesis [17,19]. The interaction between UBE2V1 and UBC13 regulates polyubiquitin chain elongation and plays a role in the activation of NF-kB [59]. The UBE2V1–UBC13 complex mediates K63-linked ubiquitination of RHBDF2, which promotes the maturation of TNFα-converting enzyme [60]. Unexpectedly, our results indicate that neither UBC13 nor members of the UBE2D family significantly affect the ubiquitination level of VHL, suggesting that UBE2V1-mediated enhancement of VHL ubiquitination occurs independently of these E2 enzymes (Fig. 6D and Fig. S8A). Intriguingly, UBE2S, which was previously reported to catalyze E3-independent K11-linked ubiquitination at residues K171 and K196 of VHL in HCC [29], collaborates with UBE2V1 in promoting K11-/K48-branched ubiquitination on VHL. These findings indicate that UBE2V1 may facilitate the synthesis of K48-linked ubiquitin chains in conjunction with UBE2S, highlighting its role in the dynamic regulation of VHL stability. Our findings reveal a previously unrecognized functional synergy between UBE2V1 and UBE2S, identifying a novel E2–E2 complex that orchestrates the ubiquitination and degradation of VHL. Nevertheless, additional research is required to fully understand the precise mechanisms underlying the functional interplay between UBE2V1 and UBE2S.

Our findings reveal a previously unrecognized positive feedback loop: HIF-1α-induced UBE2V1 expression further stabilizes HIF-1α, thereby amplifying its own activation and creating a self-reinforcing oncogenic circuit. While direct HIF-1α inhibition represents an attractive therapeutic strategy, its clinical application has faced considerable challenges due to issues such as compensatory pathways and suboptimal pharmacokinetics. This is well illustrated by the development history of PX-478, which demonstrated efficient target inhibition and a reasonable safety profile in a phase I dose-escalation study in cancer patients (NCT00522652); its clinical development has not advanced beyond early-phase trials, reflecting the broader difficulties in pharmacologically targeting HIF-1α [61]. These limitations highlight the strategic value of targeting UBE2V1 as an upstream intervention in this pathway. While no E2 enzyme-targeting therapeutics have yet received clinical approval, several candidate molecules are under active investigation across various cancer types. For example, the UBE2T inhibitor M435-1279 has shown promising preclinical efficacy in triple-negative breast cancer [62], and compound NSC697923 has demonstrated activity against UBC13 in lymphoma [63]. These developments highlight the growing recognition of this enzyme class as druggable targets. Therefore, targeted inhibition of UBE2V1 could disrupt this circuit, destabilize HIF-1α, and suppress its downstream pro-tumorigenic pathways, thereby providing a novel therapeutic strategy for hypoxic HCC that may overcome the current limitations of HIF inhibitors.

Despite providing mechanistic insights into the HIF-1α–UBE2V1–HIF-1α axis, several limitations remain to be addressed in future work: (a) Most experiments were conducted using cell lines and require further validation using patient-derived xenograft or conditional UBE2V1 knockout models; (b) the synergistic mechanisms between UBE2V1 and UBE2S remain to be fully elucidated, particularly in terms of their impact on ubiquitin chain topology; (c) although significant up-regulation of nuclear UBE2V1 was observed in HCC tissues and cell lines, its potential role in facilitating HIF-1α nuclear translocation or other nuclear functions requires further mechanistic exploration; and (d) the development of UBE2V1-specific small-molecule inhibitors and the evaluation of their therapeutic efficacy in hypoxic HCC models represent promising future directions for translational applications.

**Fig. 9.**
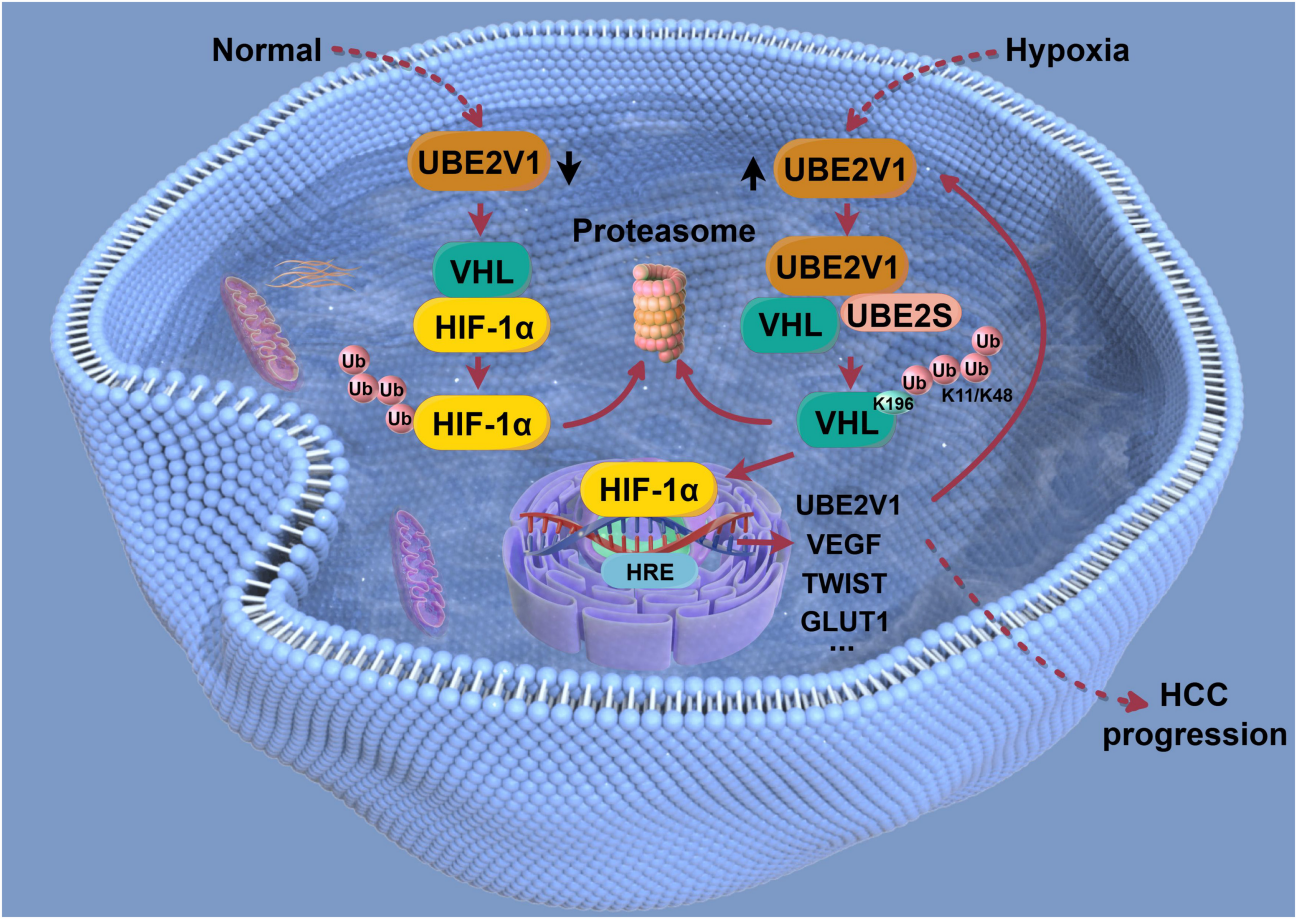
The mechanistic scheme of this study. The hypoxic microenvironment within HCC induces *UBE2V1* transcription by facilitating HIF-1α binding to a hypoxia-response element (HRE) in the UBE2V1 promoter. Up-regulated UBE2V1 then competitively binds to the VHL β-domain and, in complex with UBE2S, catalyzes K11/K48-linked ubiquitination at K196 on VHL. This impairs VHL-mediated ubiquitination and degradation of HIF-1α, leading to HIF-1α stabilization, nuclear accumulation, and transcriptional activation, ultimately driving HCC progression.

This study represents the first comprehensive characterization of the HIF-1α–UBE2V1–HIF-1α positive feedback loop as a central mechanism of hypoxia adaptation in HCC (Fig. 9). Hypoxia induces *UBE2V1* transcription through HIF-1α binding to its promoter region. Up-regulated UBE2V1 then competitively binds to the VHL β-domain and, in complex with UBE2S, catalyzes K11/K48-linked ubiquitination at K196 on VHL. This impairs VHL-mediated ubiquitination and degradation of HIF-1α, leading to HIF-1α stabilization, nuclear accumulation, and transcriptional activation, thereby driving HCC progression. This discovery not only advances our understanding of the molecular mechanisms underlying HCC progression but also provides a mechanistic foundation for novel therapeutic strategies targeting the UBE2V1–HIF-1α axis. Future translational studies should evaluate the clinical utility of this pathway both as a prognostic biomarker and as a therapeutic target, offering new avenues for improving outcomes in HCC patients.

## Methods

### Patients and clinical specimens

HCC samples and matched adjacent nontumor tissues were acquired from Zhejiang Provincial People’s Hospital. The inclusion criteria were as follows: (a) histologically confirmed primary HCC, (b) underwent surgical resection, and (c) availability of complete clinicopathological and follow-up data. The exclusion criteria included the following: (a) receipt of any preoperative anticancer therapy, (b) history of other malignancies, and (c) incomplete medical records. Clinical and demographic characteristics of the enrolled patients are summarized in Table S1.

### Cell culture

The HCC cell lines (LM3, Hep3B, Huh7, HepG2, MHCC97H, and PLC/PRF/5) and HEK-293T were purchased from the Cell Bank of the Chinese Academy of Sciences. The human hepatocyte cell line THLE-2 was procured from Jinyuan Biology. Hep3B, PLC/PRF/5, and HepG2 were cultured in minimum essential medium (MEM, VivaCell, C3060-0500); other cells were cultured in Dulbecco’s modified Eagle medium (DMEM, VivaCell, C3113-0500).

### Cell transfection and lentiviral infection

siRNAs targeting HIF-1α, UBE2S, UBE2V1, and VHL, along with the corresponding plasmid constructs and UBE2V1 lentivirus, were purchased from GenePharma (Shanghai, China). Truncation mutants of UBE2V1 and VHL, as well as luciferase reporter vectors, were obtained from Sangon Biotech (Shanghai, China). The HA-ubiquitin plasmid and its mutant variants were generated in-house. Transfection of plasmids and siRNAs was carried out using Liposomal Transfection Reagent (Yeasen, 40802ES03) or Lipofectamine 3000 (Invitrogen, L3000015). For lentiviral infection, cells were subjected to puromycin (Yeasen, 60209ES60) selection postinfection to establish stable cell lines. The siRNA and shRNA sequences utilized in this study are detailed in Table S2.

### Real-time quantitative PCR

The reagents and experimental procedures used in this study were consistent with those described in our previously established protocol [64]. The primer sequences are provided in Table S3.

### Western blot

The experiment was performed following the methodology outlined in previous studies [65]. Detailed information of primary antibodies is listed in Table S4.

### CCK-8 and colony formation assays

For CCK-8 assays, after the indicated transfections or treatments, Huh7 and MHCC97H cells were plated into 96-well plates at appropriate densities. After cell adhesion, CCK-8 reagent (Yeasen, 40203ES80) was added at the corresponding time point. Proliferation rates were calculated relative to the 0 h time point. Cells were seeded at low density and grown in complete medium for colony formation assays. After 7 to 14 days, colonies were washed with phosphate-buffered saline (PBS), fixed with 4% paraformaldehyde, and stained with 2% crystal violet. The plates were rinsed with distilled water and air-dried. Images were taken for documentation.

### Wound healing and transwell assays

Wound healing and transwell assays were performed to assess the migratory ability of tumor cells, using the experimental approach and protocols detailed in our earlier published study [65].

### Tumor sphere formation assay

HCC cells were plated in 6-well plates with an ultralow attachment surface to prevent cell adhesion. The culture medium was composed of serum-free DMEM/F12 (VivaCell, C3130) and enriched with recombinant human basic fibroblast growth factor (Peprotech, 100-18B), N-2 supplement (Thermo Fisher Scientific, 17502048), and animal-component-free recombinant human epidermal growth factor (Peprotech, AF-100-15).

### Co-immunoprecipitation

Total protein extracts were obtained by lysing cells in IP lysis buffer. The resulting cell lysate was mixed with the primary antibody at 4°C for overnight rotation. The supernatant was gently discarded, and Protein A/G Magnetic Beads (Selleck, B23201) were introduced at ambient temperature for a 15-min incubation period. The magnetic bead-bound complexes were then subjected to 3 rounds of washing with washing buffer. The complexes were mixed using 1× loading buffer and subjected to heating at 95°C for 10 min to elute the immunoprecipitated proteins. Subsequently, the magnetic beads were then isolated using a magnetic separator, and the purified protein samples were subjected to WB analysis for detection of the target protein.

### Immunofluorescence

Following cell adherence, the samples were rinsed 3 times with PBS and then fixed using paraformaldehyde. Blocking was performed using QuickBlock blocking buffer (Beyotime, P0260). The primary antibodies were added and incubated at 4 °C overnight. Afterward, unbound antibodies were washed away with 3 rinses of PBS. Fluorescently labeled secondary antibodies were then applied for incubation. The cell nuclei were stained with DAPI solution (Beyotime, P0131) to ensure the stability of fluorescence signals.

For the colocalization analysis of UBE2V1, UBE2S, and VHL, multiplex fluorescent IHC was performed using the IRIS Kit CmTSA Kit (HUABIO, 900803) according to the manufacturer’s instructions. This method enabled sequential staining and visualization of the 3 proteins in the same sample.

### Chromatin immunoprecipitation

ChIP assays were conducted on cells using the ChIP assay kit (Beyotime, P2078) in accordance with the manufacturer’s protocol. In brief, protein–DNA cross-links are generated by formaldehyde treatment of cells, after which the reaction is stopped through the addition of glycine. After cell lysis, chromatin was extracted and fragmented into 200- to 800-bp fragments via sonication to enhance immunoprecipitation efficiency. The resulting chromatin lysates were incubated with either anti-HIF-1α antibody to enrich DNA sequences specifically bound by HIF-1α, or normal rabbit IgG as a negative control to evaluate background binding levels. Immune complexes were captured using beads, and the cross-links were reversed under high-temperature conditions to release the associated DNA. Purified DNA was subsequently collected for downstream analysis. RT-qPCR was performed with primers specific to the target gene, which were designed to amplify the region of interest. The relative enrichment was determined by comparing the Ct values from the anti-HIF-1α immunoprecipitated samples with those from the IgG control group.

### Immunohistochemistry

All tissue samples underwent fixation, paraffin embedding, and sectioning, followed by antigen retrieval and endogenous peroxidase blocking. After an overnight incubation with the primary antibody, the samples were washed with PBS and then exposed to the secondary antibody for a 1-h incubation. Immunocomplexes were detected using a DAB staining kit (ZSGB-BIO, PV8000) with a chromogenic reaction time of 5 min. The tissue slices were subsequently counterstained with hematoxylin, dried with graded alcohol, cleaned with xylene, and mounted with neutral balsam for digital pathological imaging. The staining results were independently assessed by 2 pathologists based on the intensity and extent of staining, and any discrepancies were settled through joint reevaluation under identical microscopic conditions.

### Dual luciferase reporter assay

Huh7 and MHCC97H cells were cotransfected with the UBE2V1-luc reporter plasmid, HIF-1α expression vector, and the pRL-TK control construct. Following a 48-h incubation, cells were collected and lysed, and luciferase activity was determined using the Dual-Luciferase Reporter Assay Kit (Beyotime, RG027).

### Mouse models

Female BALB/c nude mice (5 to 6 weeks old) were obtained from Hangzhou Medical College. MHCC97H cells with stable UBE2V1 knockdown or overexpression were administered via subcutaneous or intravenous injection. For subcutaneous tumor implantation, a total of 35 mice were randomly assigned to 7 experimental groups (*N* = 5 per group), with each animal receiving 4 × 10^6^ cells. Utilize a digital caliper to measure the length (*L*) and breadth (*W*) of the tumor, subsequently calculating the tumor volume (*V*) with the formula *V* = 0.52 × *L* × *W*^2^. In the intravenous injection model, 28 mice were randomly allocated to 7 groups, with 4 mice per group, with each mouse receiving 5 × 10^6^ cells via tail vein injection. Following tumor establishment, PX-478 was administered intraperitoneally every 2 days at dosages of 20 or 40 mg/kg. These intermediate doses were selected based on established preclinical regimens (20 mg/kg daily; 50 mg/kg every 3 days) to balance efficacy and tolerability in our model [4,29]. At the endpoint of the experiment, animals were euthanized humanely, and tumors and lungs were excised for determination of volume and weight.

### RNA sequencing

Three independent biological replicates of Huh7 cell samples were collected under normoxic and hypoxic conditions. Total RNA was isolated utilizing TRIzol reagent. DNA nanoballs were then loaded onto a patterned nanoarray, and paired-end sequencing with 150-base-pair read lengths was conducted on the G400 sequencing platform (BGI-Shenzhen, China). Transcriptome assembly was carried out with Trinity (v2.13.2), and the completeness of the assembled transcriptome was evaluated using BUSCO. Processed reads were aligned to the assembled transcript sequences utilizing Bowtie2 (v2.4.5), and gene expression levels were estimated using RSEM (v1.3.1). Gene expression differences among experimental groups were assessed using DESeq2 (v1.48.1), applying a significance threshold of *P* < 0.05. A pheatmap heatmap showed hierarchical clustering of gene expression differences. Functional classification of differentially expressed genes was conducted using GO and KEGG annotation data, in conjunction with standardized classification systems.

### Mass spectrometry

Protein extracts were isolated from cultured cell lines and subsequently separated by gel electrophoresis. Distinct protein bands were removed from the gel and treated with trypsin in an ammonium bicarbonate solution. After digestion, the generated peptide mixtures were fractionated by chromatography and subjected to analysis on a timsTOF Pro mass spectrometer (Bruker Daltonics Inc).

### Bioinformatics analysis of public datasets

Gene expression data from the GTEx project and TCGA-LIHC cohort were downloaded using UCSC Xena. The microarray datasets GSE76427, GSE54236, and GSE155505 were retrieved from the GEO database. RNA-seq data across multiple tumor types were retrieved from the TIMER2.0 database. The promoter base sequence of UBE2V1 was obtained from the NCBI gene database, and the potential binding motif of HIF-1α within the UBE2V1 promoter region was predicted using the JASPAR 2024 database. Information regarding ubiquitin-related genes and VHL-mediated ubiquitination sites was collected from the iUUCD database.

### Molecular docking model

The 3-dimensional structures of UBE2V1 (UniProt: Q13404) and VHL (UniProt: P40337) were predicted using AlphaFold3 based on their canonical amino acid sequences retrieved from the UniProt database. The AlphaFold3 server (https://alphafoldserver.com/) was employed for both monomer structure prediction and complex docking. Protein–protein interaction interfaces were analyzed with PyMOL (version 3.0), considering residues within 5 Å as potentially interacting.

### Biologically active compound

CoCl_2_ (Sigma-Aldrich, 232696) was applied to induce chemical hypoxia in cells. To investigate protein stability and regulatory mechanisms, stable HCC cell lines were treated with CHX (Selleck, S7418), a protein synthesis inhibitor; MG132 (Selleck, S2619), a proteasome inhibitor; PX-478 (MCE, HY-10231), a selective HIF-1 inhibitor; and NSC697923 (MCE, HY-13811), an inhibitor of UBC13. After drug exposure, cellular samples were harvested and processed for RT-qPCR or WB analysis to assess molecular changes.

### Statistical analysis

All sequencing data were processed using R software (v4.2.2), and statistical analyses were performed with GraphPad Prism 8. The relationship between UBE2V1 expression levels and clinicopathological characteristics was evaluated using the chi-square test and Fisher’s exact test. Kaplan–Meier survival analysis, together with the log-rank test, was employed to evaluate differences in OS between groups. Given that multiple endpoints were simultaneously examined, the *P* values were adjusted using the Holm–Bonferroni correction method. Univariate and multivariate Cox proportional hazards regression models were utilized to determine the prognostic relevance of various clinical and pathological variables. In univariate analysis, variables were independently assessed with Holm–Bonferroni correction to control type I error. The multivariate Cox model integrates all variables into one unified framework. Multiplicity adjustment is unnecessary due to inherent accounting of predictor interdependencies. Unadjusted *P* values are reported in the multivariate analysis. Pearson correlation analysis was performed to assess the linear relationships among the expression levels of HIF-1α and UBE2V1. For comparisons between 2 groups, a 2-tailed Student’s *t* test was applied, whereas 1-way analysis of variance (ANOVA) was used when comparing more than 2 groups.

## Ethical Approval

This study constitutes a retrospective analysis of human samples and clinical data. We have obtained an exemption from informed consent approval (No. QT2023321) from the Ethics Committee of Zhejiang Province. The animal experimentation protocol has been approved by the Animal Welfare and Ethics Committee of Zhejiang Laboratory Animal Center (Approval No. ZJCLA-IACUC-20010987).

## Data Availability

The datasets supporting the study’s findings can be requested from the corresponding authors under reasonable conditions.
