# Supplementary material for: UBE2V1 Promotes Hepatocellular Carcinoma Progression by Forming a Positive Feedback Loop with HIF-1α
Source: Research (Wash D C). 2025 Dec 23;8:1041. doi: 10.34133/research.1041 (PMC12722638; doi:10.34133/research.1041)

A

VHL ubiquitin molecular modification site

Sequence	Modification sites
RCLQVVRSLVKPENYRRLDIV	K171
EDLEDHPNVQKDLERLTQERI	K196
ANITLPVYTLKERCLOVVRSL	K159

B

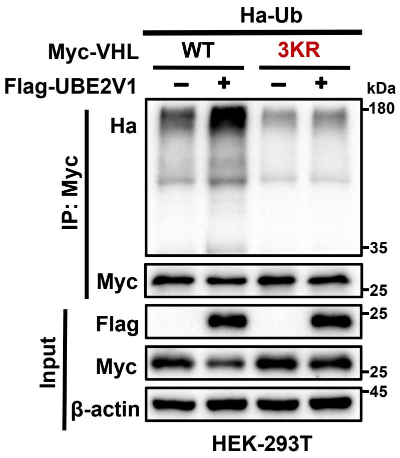

C

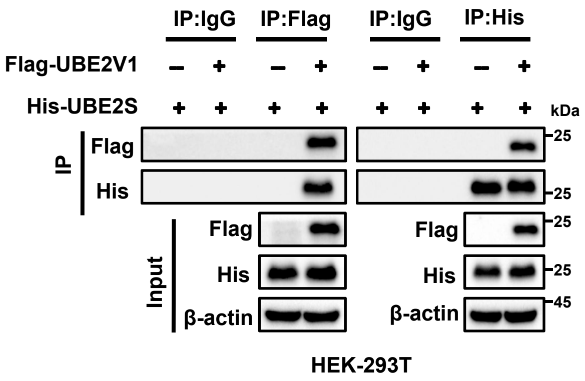

D

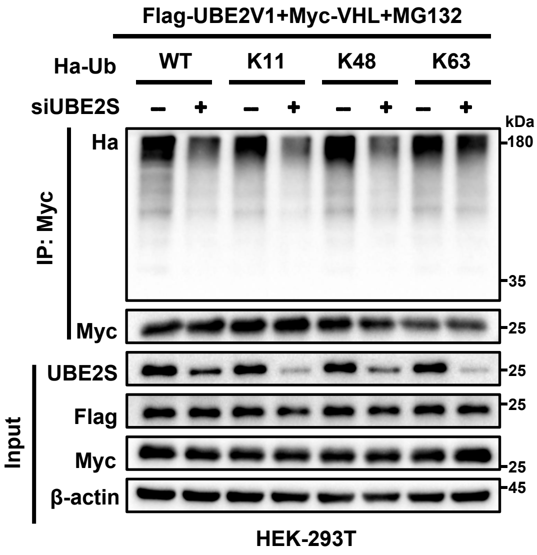

Supplement: Supplementary 1 — Figs. S1 to S8 Tables S1 to S4 [file research.1041.f1.zip › Fig S5.pdf]
