# Supplementary material for: UBE2V1 Promotes Hepatocellular Carcinoma Progression by Forming a Positive Feedback Loop with HIF-1α
Source: Research (Wash D C). 2025 Dec 23;8:1041. doi: 10.34133/research.1041 (PMC12722638; doi:10.34133/research.1041)
Supplement: Supplementary 1 — Figs. S1 to S8 Tables S1 to S4 [file research.1041.f1.zip › Revised Supplementary Figures clear version.docx]

**
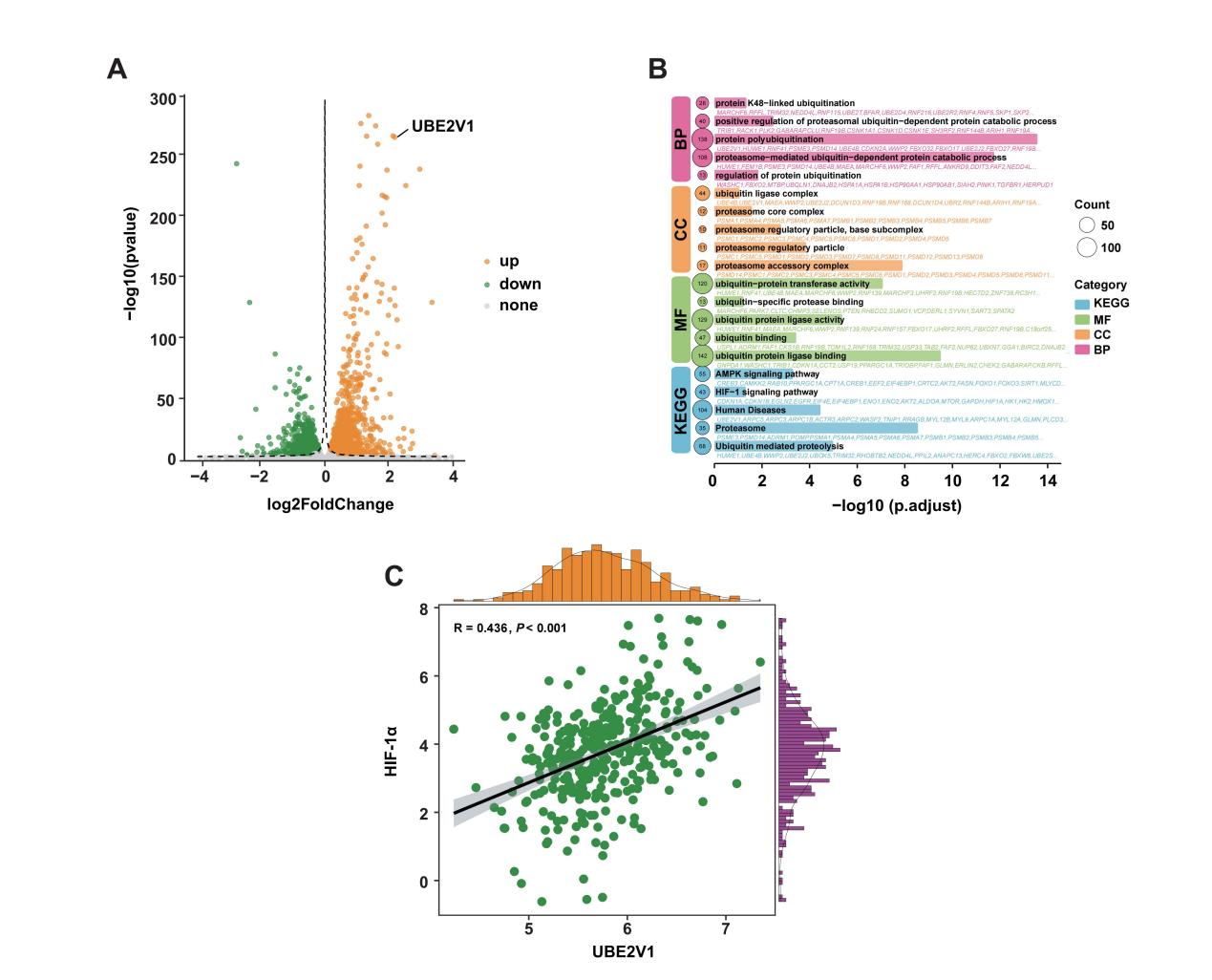
**

**Fig. S1.** Differential gene expression analysis in HCC under hypoxia versus normoxia. (A) The volcano plot of significantly dysregulated genes in hypoxic HCC cells. (B) Enrichment analysis highlights ubiquitination-related signaling pathways. (C) Correlation analysis between UBE2V1 and HIF-1α mRNA expression in the TCGA-LIHC cohort (n=367, Pearson).


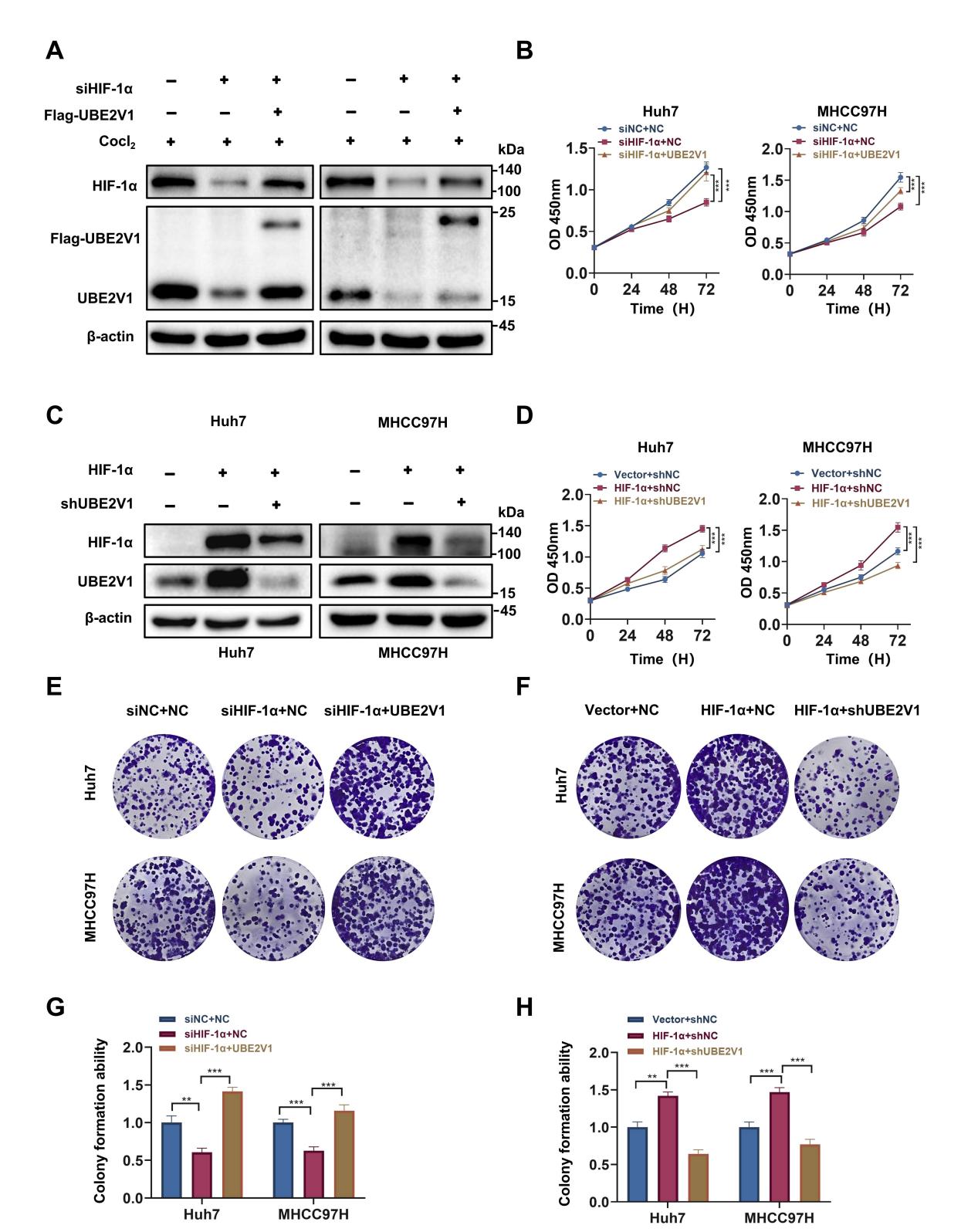


**Fig. S2.** UBE2V1 contributes to the regulation of HIF-1α in the malignant progression of HCC. (A) WB analysis of protein expression after UBE2V1 overexpression and HIF-1α knockdown under hypoxic conditions. (B) CCK-8 assays showing that UBE2V1 overexpression partially rescues the impaired proliferation induced by HIF-1α knockdown (n=3, one-way ANOVA). (C) WB analysis of protein expression following UBE2V1 knockdown and HIF-1α overexpression. (D) CCK-8 assays demonstrating that UBE2V1 knockdown inhibits HIF-1α overexpression-induced proliferation (n=3, one-way ANOVA). (E to H) Colony formation assays further validated the differences in cell proliferation potential between experimental groups (n=3, one-way ANOVA). **p* < 0.05, ******p* < 0.01, *******p* < 0.001, ********p* < 0.0001 (B, D, G, H: n=3, one-way ANOVA).


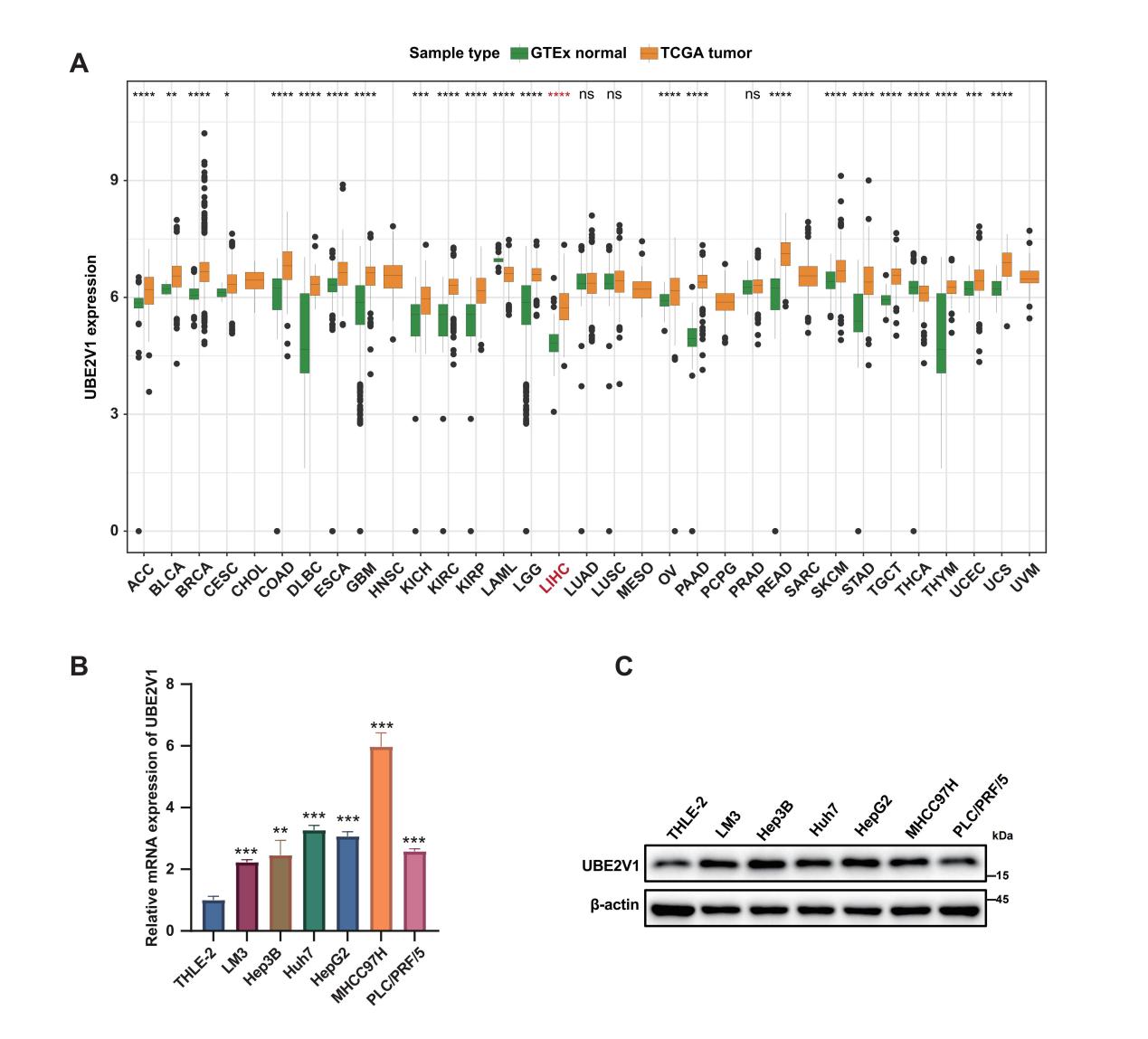


**Fig. S3.** Comprehensive analysis of UBE2V1 expression profile in pan-cancer and HCC Lines. (A) Differential expression analysis of UBE2V1 across pan-cancer types. The bar chart depicts the relative mRNA expression levels of UBE2V1 in GTEx normal tissues (green) compared to TCGA tumor tissues (yellow). (B) RT-qPCR analysis of UBE2V1 mRNA expression in the normal hepatocyte cell line THLE-2 and HCC lines (n=3, one-way ANOVA). (C) WB analysis of UBE2V1 protein.


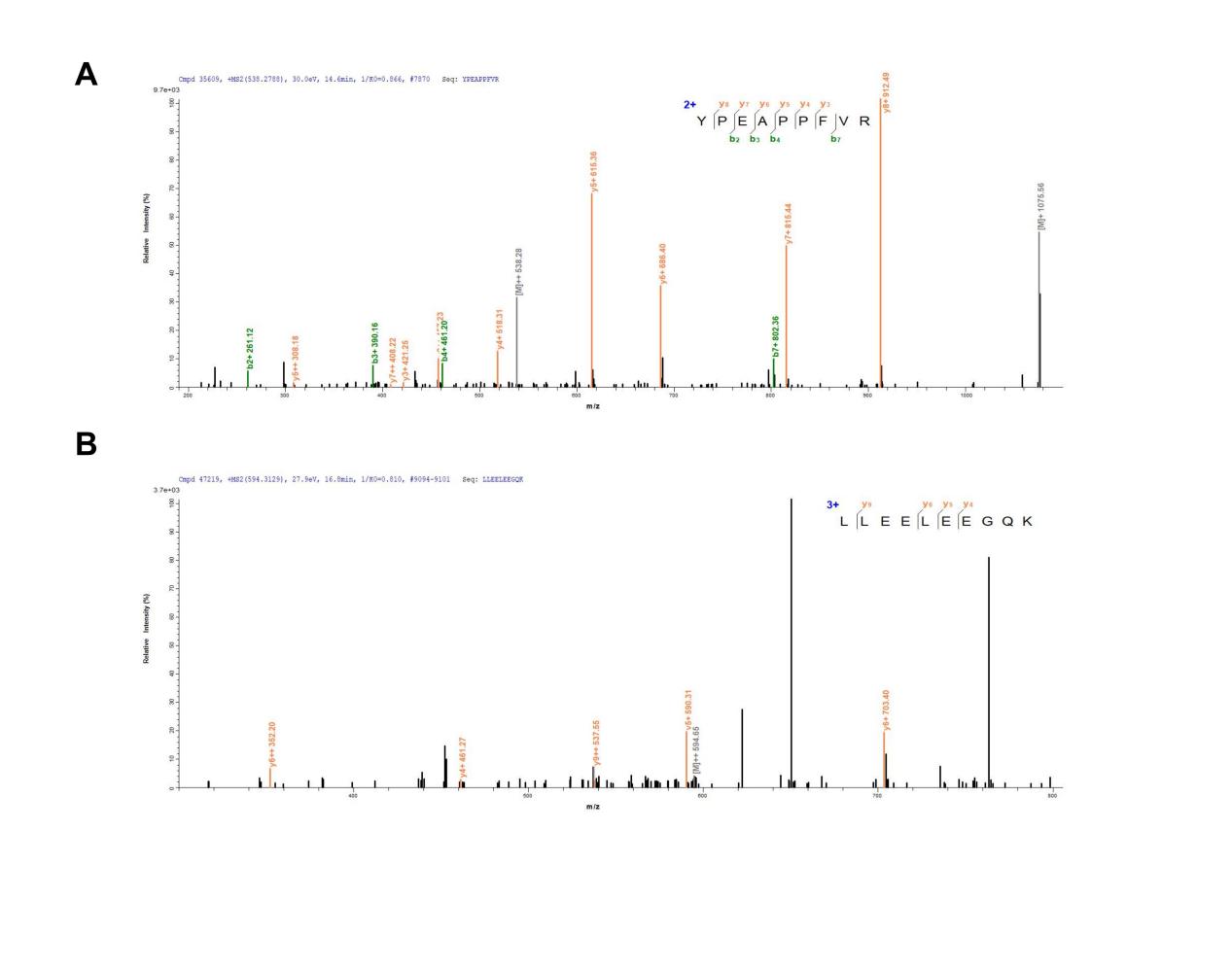


**Fig. S4.** Tandem mass spectrum of the VHL peptide segment. (A) The UBE2V1 peptide YPEAPPFVR. (B) The UBE2V1 peptide LLEELEEGQK.


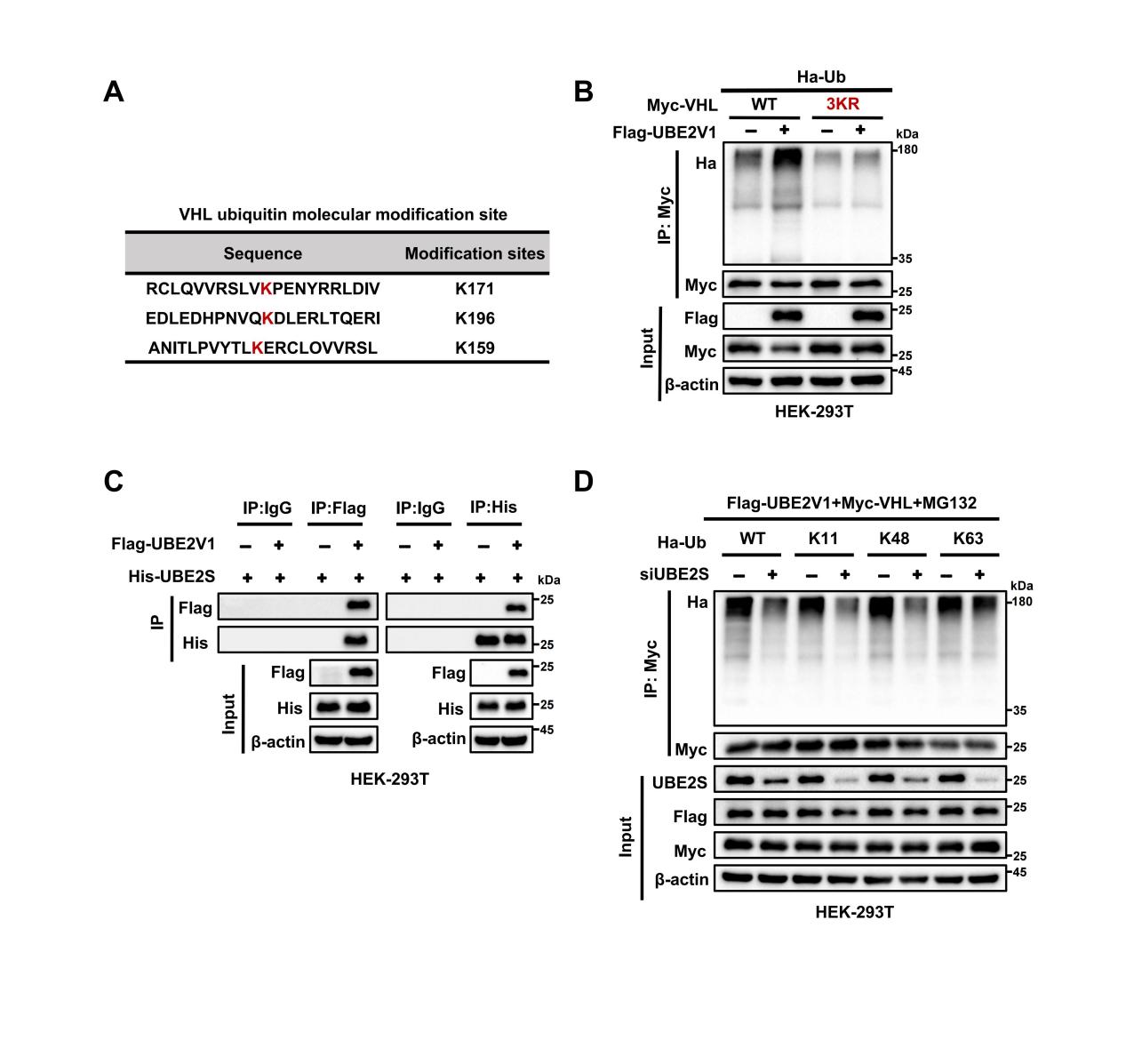


**Fig. S5.** UBE2V1 promotes K11- and K48-Linked polyubiquitination of VHL in complex with UBE2S. (A) Bioinformatic analysis of potential ubiquitination sites in the VHL protein. The table presents amino acid sequences encompassing K159, K171, and K196. (B) Co-transfection of Flag-UBE2V1, HA-Ub, Myc-VHL, or Myc-VHL 3KR (K159, K171, K196 all mutated to arginine) plasmids into HEK-293T cells to assess the impact of multiple lysine mutations on ubiquitination. (C) Confirmation of the interaction between UBE2V1 and UBE2S in HEK-293T cells. (D) Specific regulation of VHL ubiquitin chain types by UBE2S knockdown in HEK-293T cells.

**
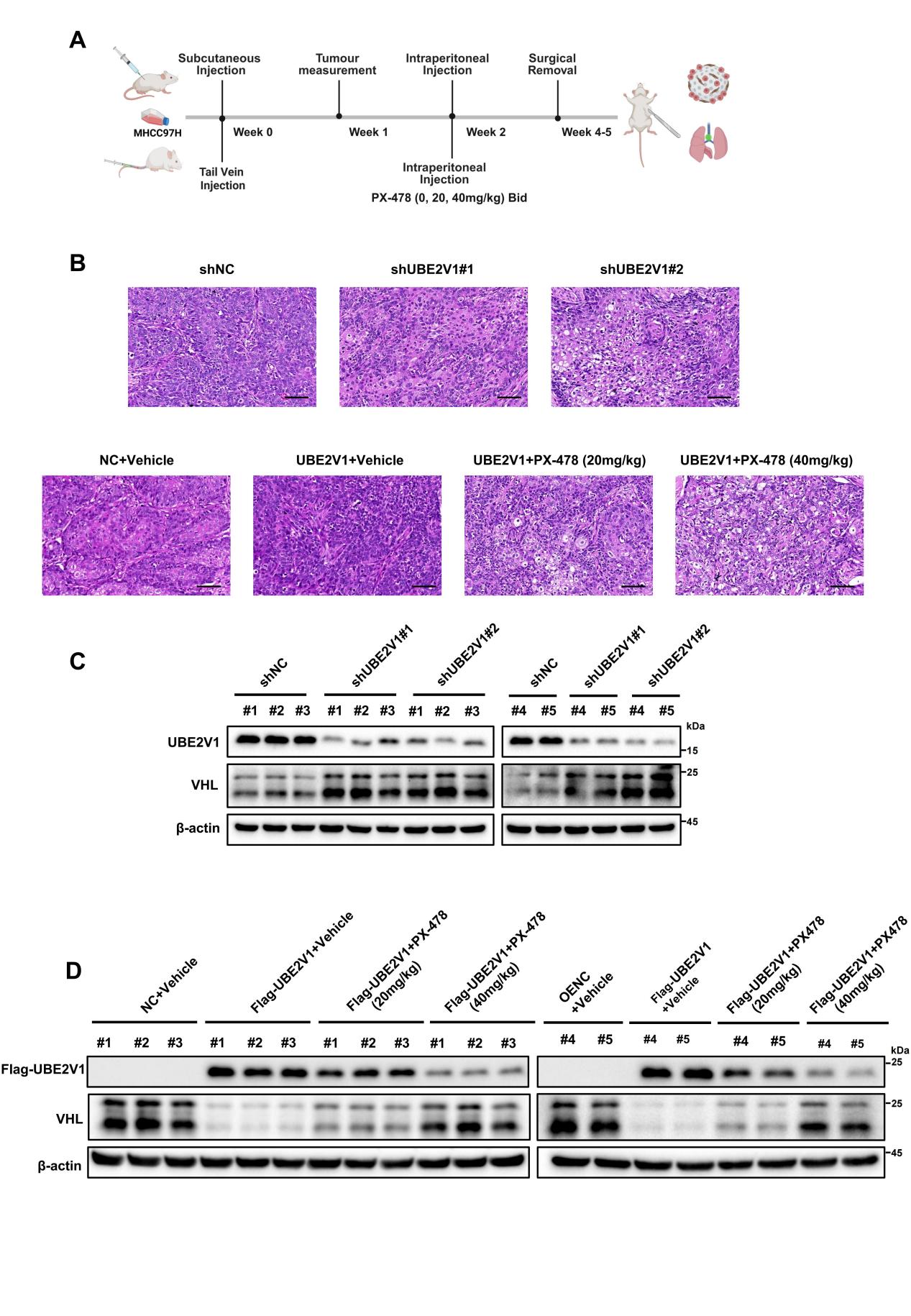
**

**Fig. S6.** Establishment and evaluation of subcutaneous tumorigenesis and tail vein metastasis models. (A) Schematic diagram illustrating the in vivo experimental procedures in nude mice. Upper panel: Tumor formation model established by subcutaneous injection of 4x10⁶ MHCC97H cells; Lower panel: Metastasis model induced by intravenous injection of 5x10^6^ MHCC97H cells via the tail vein. (B) H&E staining results of tumor tissues. (C and D) WB analysis of UBE2V1 and VHL protein expression levels in tumor tissues from nude mice. Scale bars: 100 μm.

**
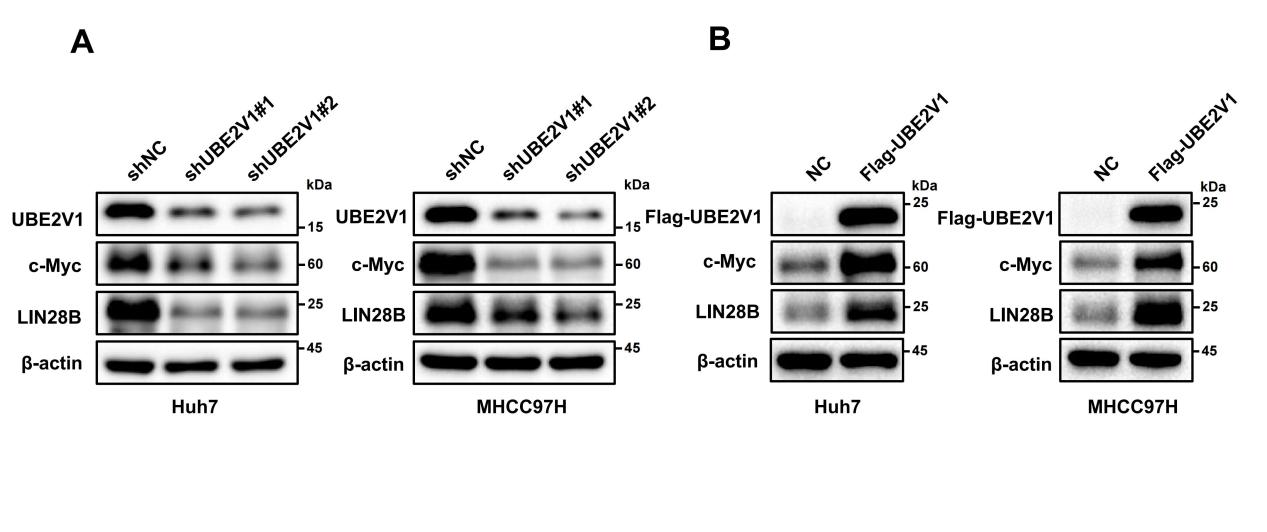
**

**Fig. S7.** UBE2V1 modulates the expression of c-Myc and LIN28B. WB analysis of UBE2V1, c-Myc, and LIN28B expression following knockdown (A) or overexpression (B) of UBE2V1.

**
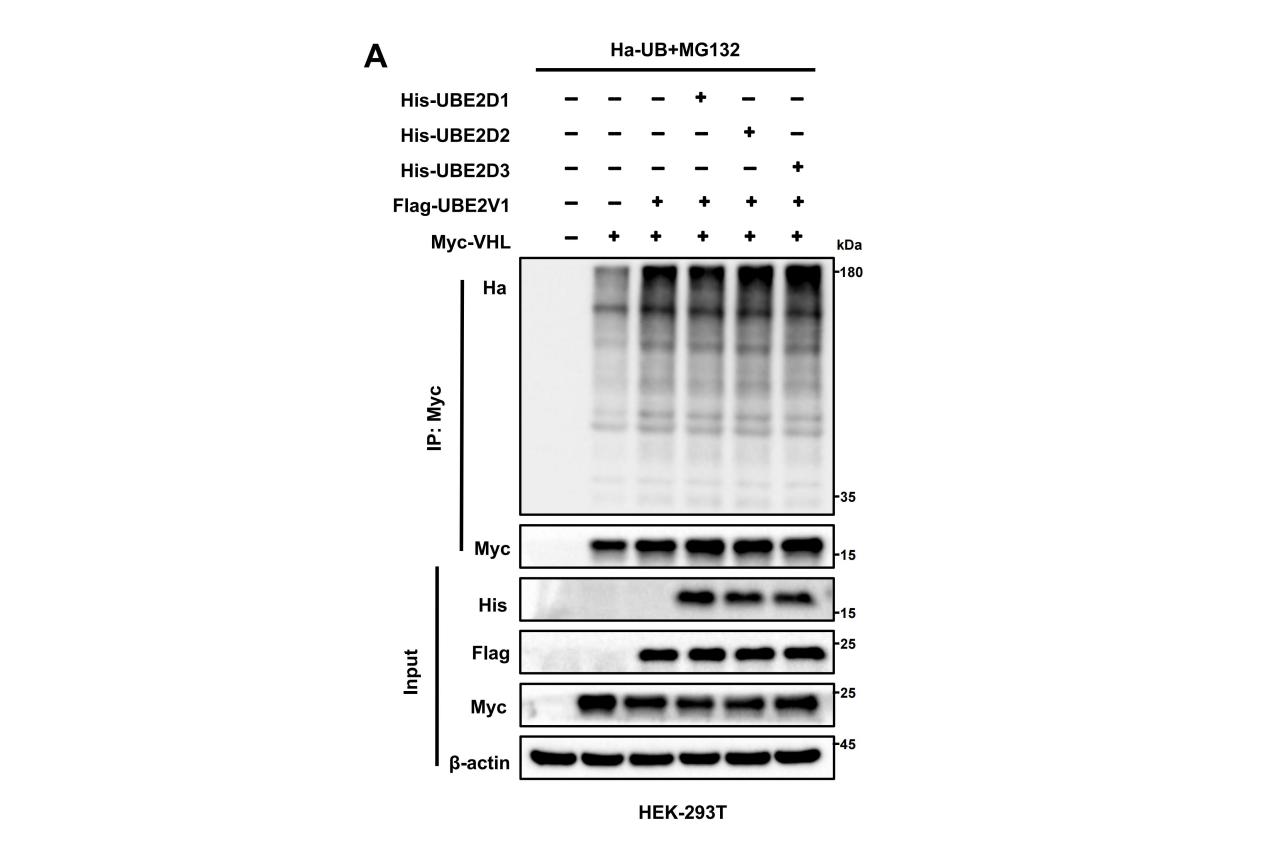
**

**Fig. S8.** Overexpression of UBE2D family members does not affect UBE2V1-induced VHL ubiquitination. (A) HEK-293T cells were co-transfected with Flag-UBE2V1, Myc-VHL, HA-Ub, and overexpression plasmids targeting His-UBE2D1, His-UBE2D2, or His-UBE2D3, as indicated. Ubiquitination levels of VHL were assessed by IP and WB.
