# Supplementary figures and images for: UBE2V1 Promotes Hepatocellular Carcinoma Progression by Forming a Positive Feedback Loop with HIF-1α
Source: Research (Wash D C). 2025 Dec 23;8:1041. doi: 10.34133/research.1041 (PMC12722638; doi:10.34133/research.1041)

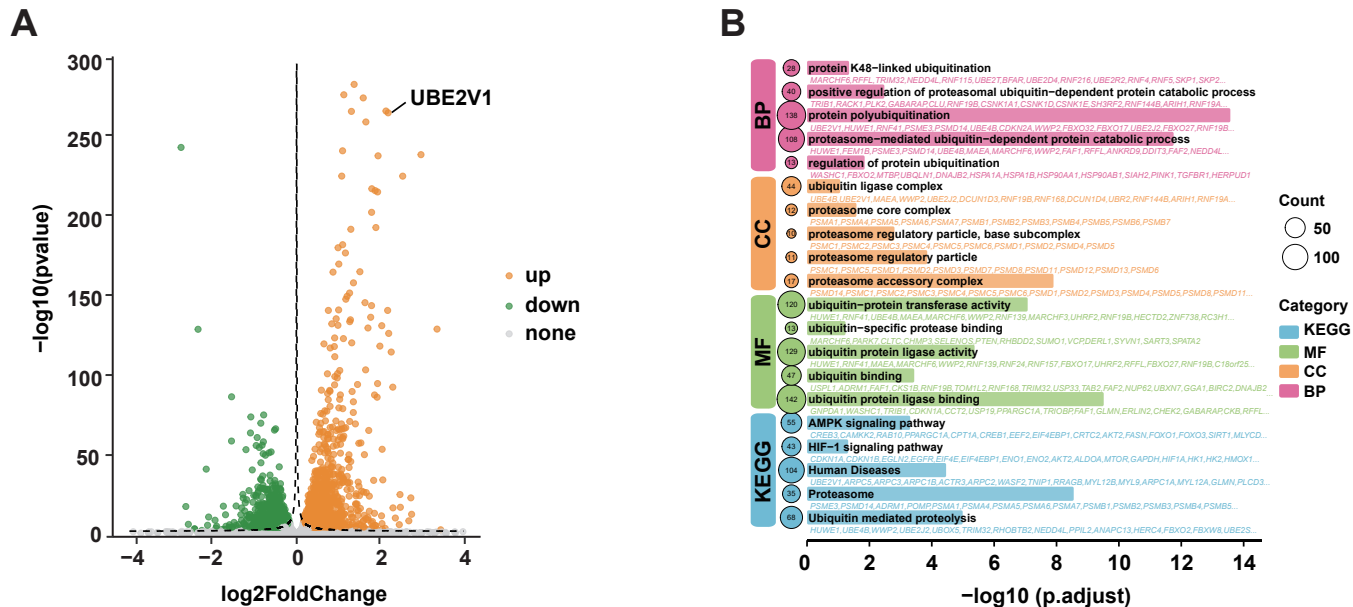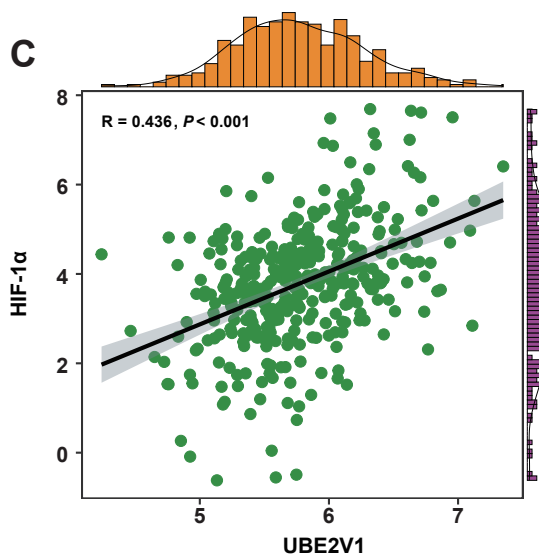

Supplement: Supplementary 1 — Figs. S1 to S8 Tables S1 to S4 [file research.1041.f1.zip › Fig S1.pdf]

**A**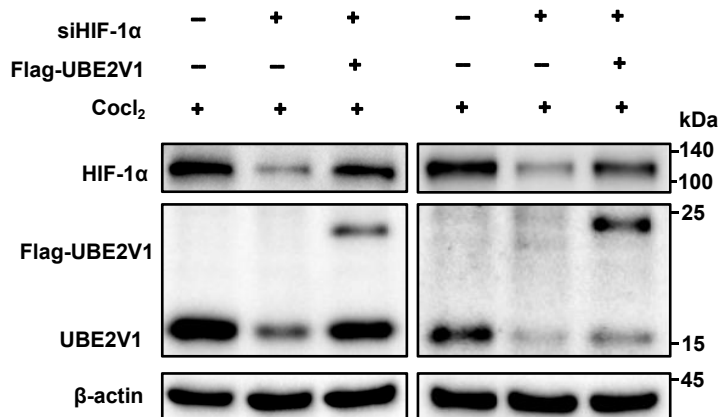**B**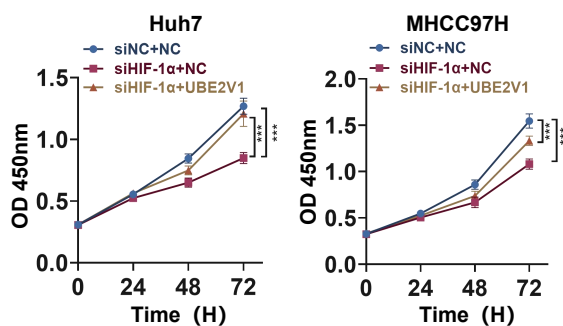**C**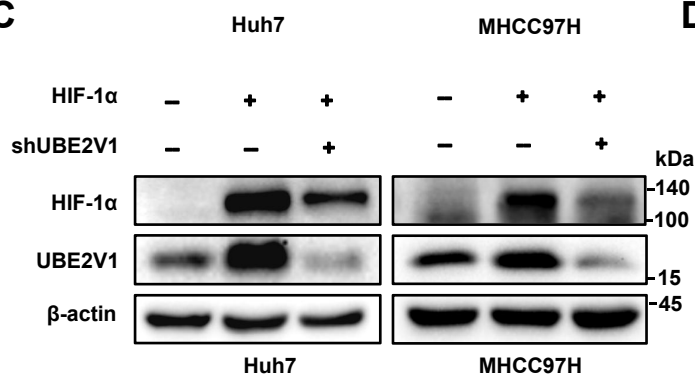**D**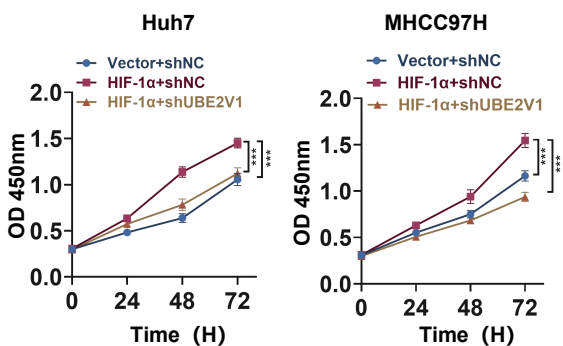**E**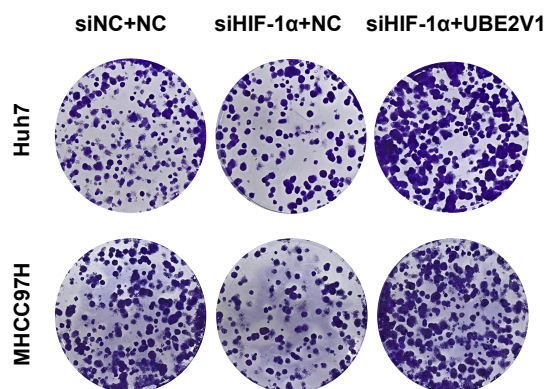**F**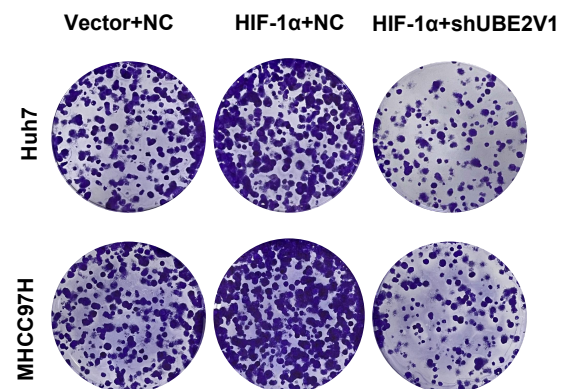**G**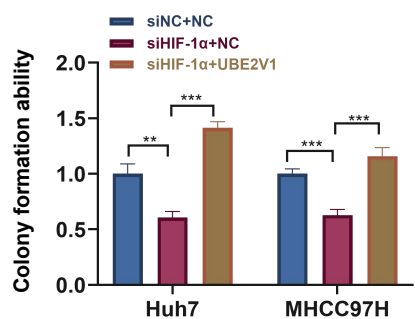**H**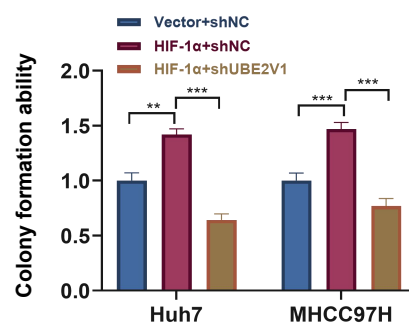

Supplement: Supplementary 1 — Figs. S1 to S8 Tables S1 to S4 [file research.1041.f1.zip › Fig S2.pdf]

**A**

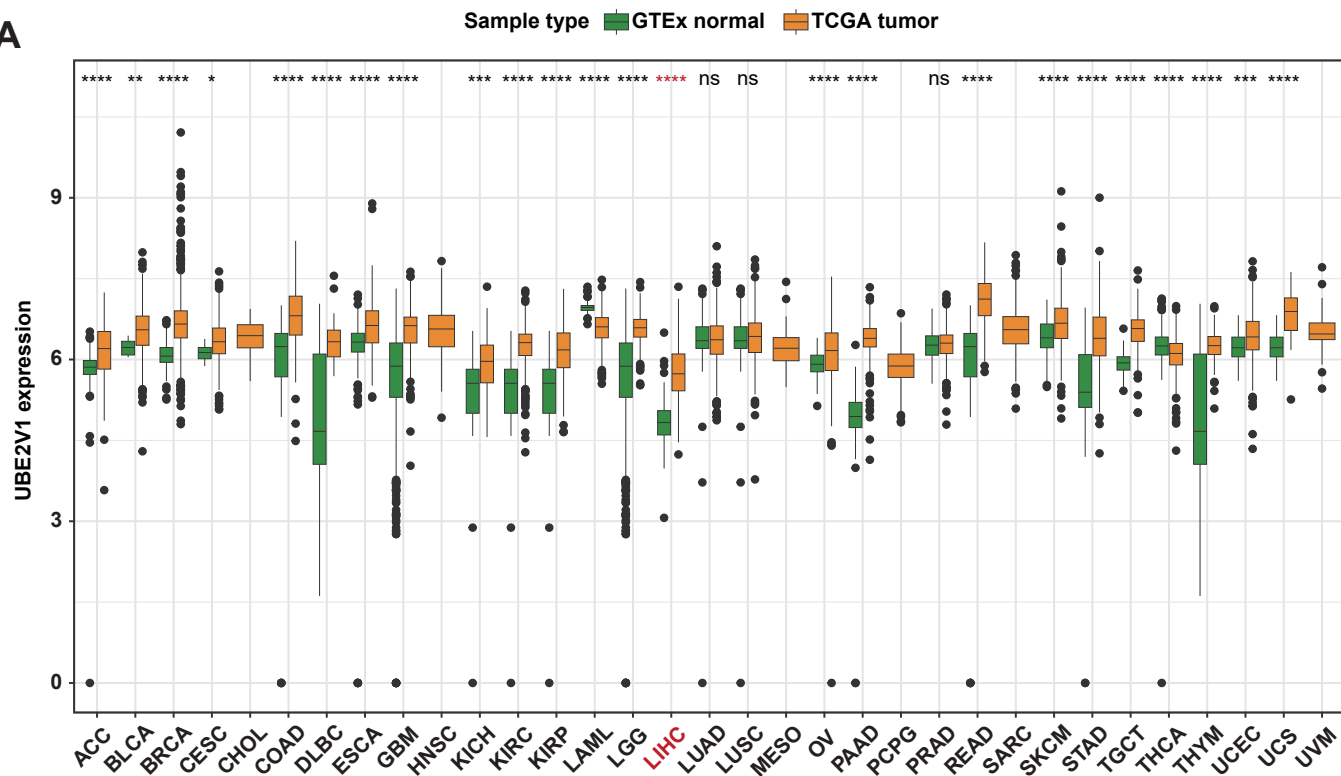

# B

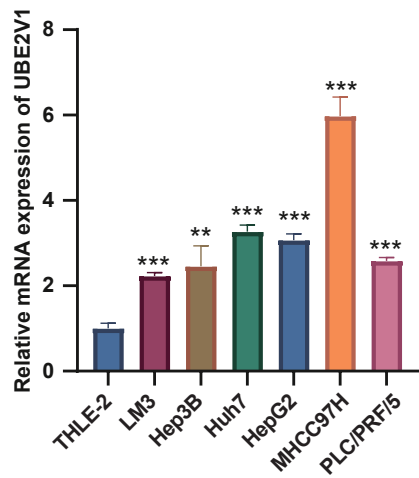

**C**

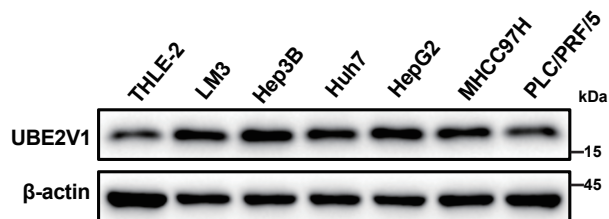

Supplement: Supplementary 1 — Figs. S1 to S8 Tables S1 to S4 [file research.1041.f1.zip › Fig S3.pdf]

**A**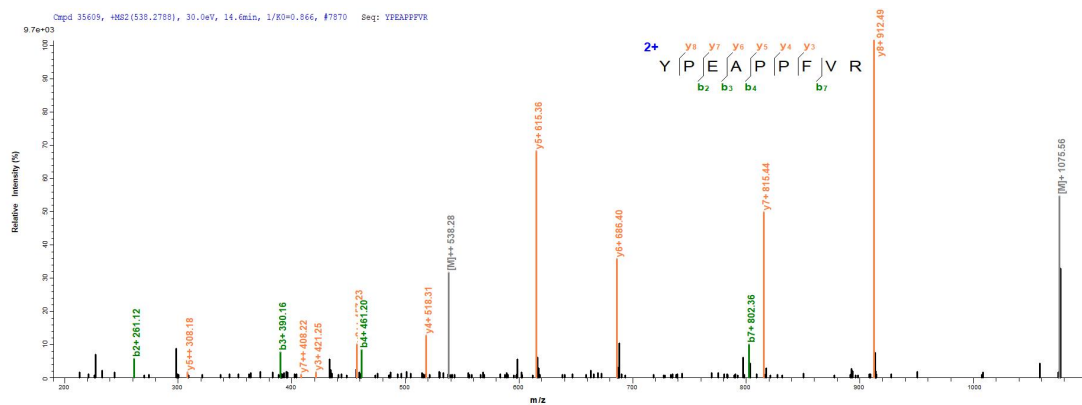**B**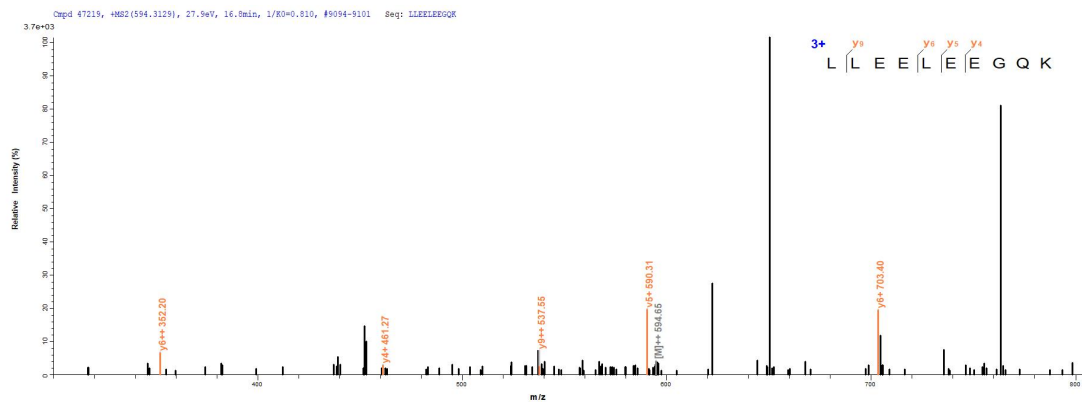

Supplement: Supplementary 1 — Figs. S1 to S8 Tables S1 to S4 [file research.1041.f1.zip › Fig S4.pdf]

**A**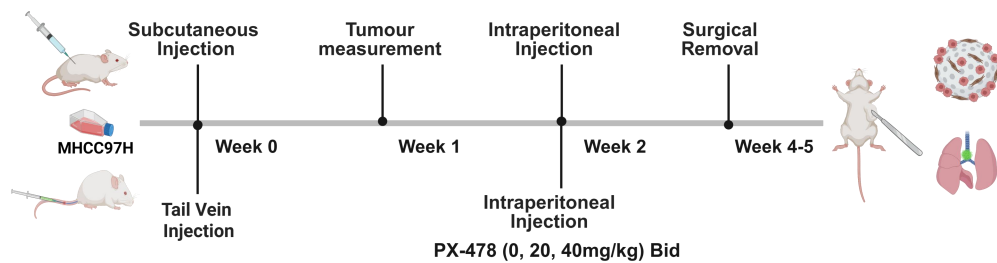**B**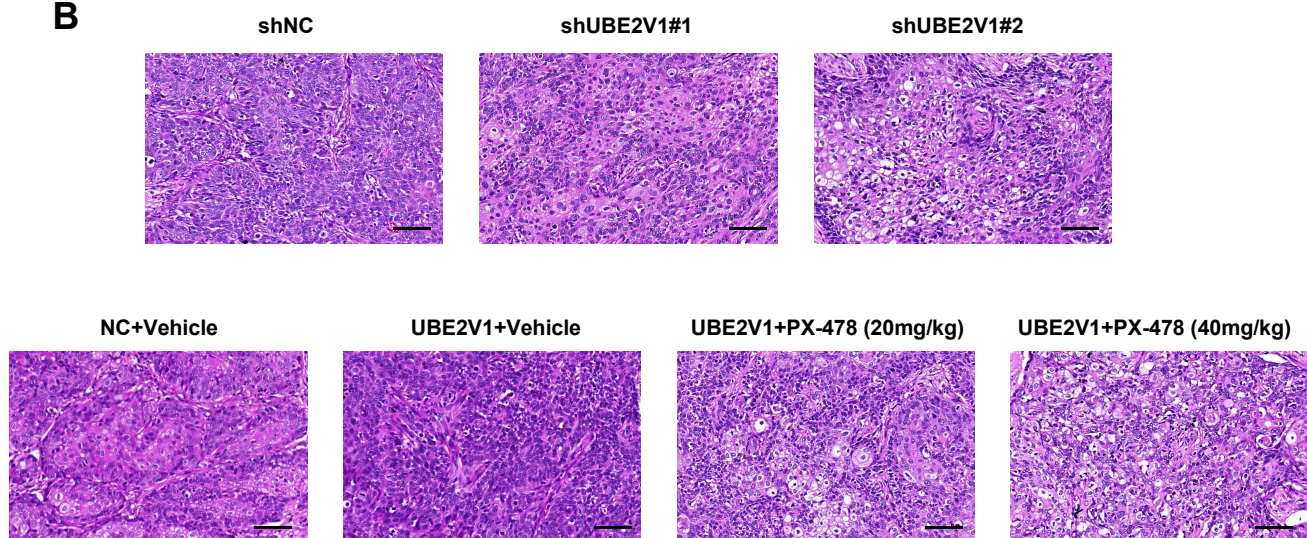**C**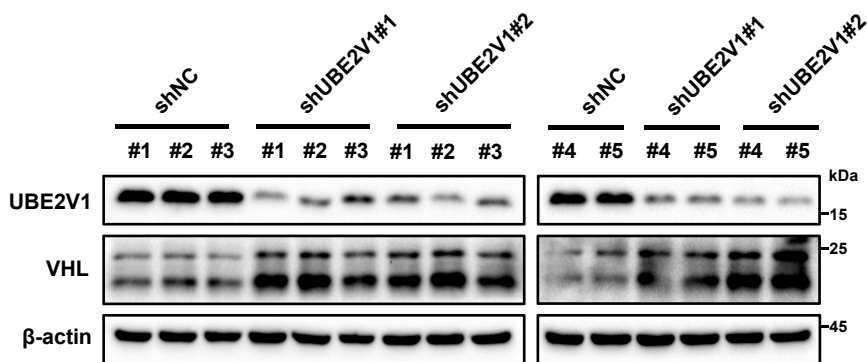**D**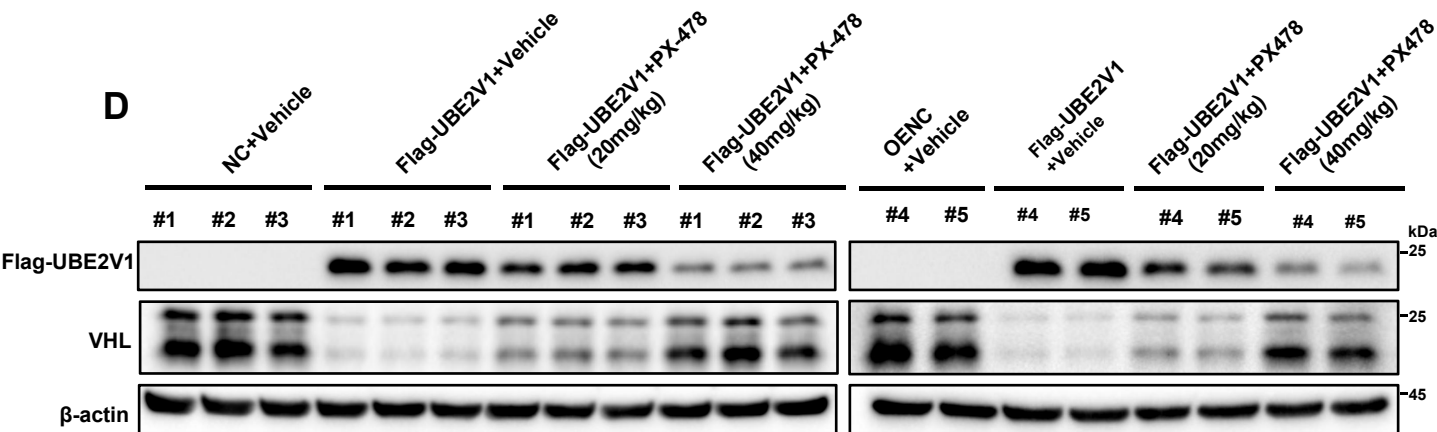

Supplement: Supplementary 1 — Figs. S1 to S8 Tables S1 to S4 [file research.1041.f1.zip › Fig S6.pdf]

**A**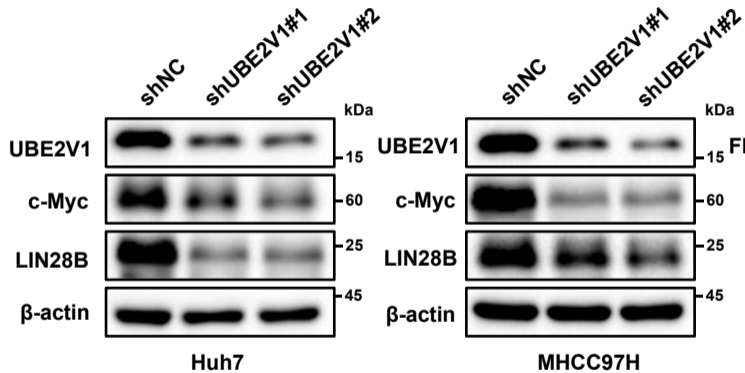**B**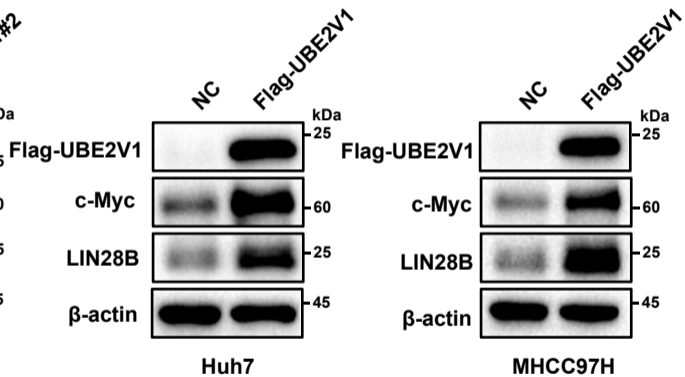

Supplement: Supplementary 1 — Figs. S1 to S8 Tables S1 to S4 [file research.1041.f1.zip › Fig S7.pdf]

**A**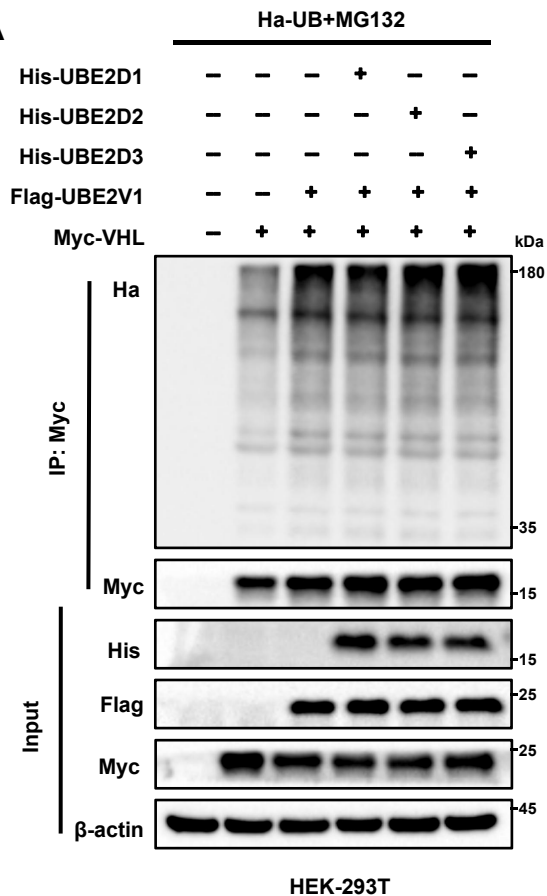

Supplement: Supplementary 1 — Figs. S1 to S8 Tables S1 to S4 [file research.1041.f1.zip › Fig S8.pdf]
